# deepNIR: Datasets for Generating Synthetic NIR Images and Improved Fruit Detection System Using Deep Learning Techniques

**DOI:** 10.3390/s22134721

**Published:** 2022-06-22

**Authors:** Inkyu Sa, Jong Yoon Lim, Ho Seok Ahn, Bruce MacDonald

**Affiliations:** 1 CSIRO Data61, Robot Perception Team, Robotics and Autonomous Systems Group, Brisbane 4069, Australia; 2 CARES, Department of Electrical, Computer and Software Engineering, University of Auckland, Auckland 1010, New Zealand; jy.lim@auckland.ac.nz (J.Y.L.); hs.ahn@auckland.ac.nz (H.S.A.); b.macdonald@auckland.ac.nz (B.M.)

**Keywords:** dataset, synthetic infrared image generation, generative adversarial network, fruit detection, object detection

## Abstract

This paper presents datasets utilised for synthetic near-infrared (NIR) image generation and bounding-box level fruit detection systems. A high-quality dataset is one of the essential building blocks that can lead to success in model generalisation and the deployment of data-driven deep neural networks. In particular, synthetic data generation tasks often require more training samples than other supervised approaches. Therefore, in this paper, we share the NIR+RGB datasets that are re-processed from two public datasets (i.e., nirscene and SEN12MS), expanded our previous study, deepFruits, and our novel NIR+RGB sweet pepper (capsicum) dataset. We oversampled from the original nirscene dataset at 10, 100, 200, and 400 ratios that yielded a total of 127 k pairs of images. From the SEN12MS satellite multispectral dataset, we selected Summer (45 k) and All seasons (180k) subsets and applied a simple yet important conversion: digital number (DN) to pixel value conversion followed by image standardisation. Our sweet pepper dataset consists of 1615 pairs of NIR+RGB images that were collected from commercial farms. We quantitatively and qualitatively demonstrate that these NIR+RGB datasets are sufficient to be used for synthetic NIR image generation. We achieved Frechet inception distances (FIDs) of 11.36, 26.53, and 40.15 for nirscene1, SEN12MS, and sweet pepper datasets, respectively. In addition, we release manual annotations of **11** fruit bounding boxes that can be exported in various formats using cloud service. Four newly added fruits (blueberry, cherry, kiwi and wheat) compound 11 novel bounding box datasets on top of our previous work presented in the deepFruits project (apple, avocado, capsicum, mango, orange, rockmelon and strawberry). The total number of bounding box instances of the dataset is 162 k and it is ready to use from a cloud service. For the evaluation of the dataset, Yolov5 single stage detector is exploited and reported impressive mean-average-precision, ${\mathrm{mAP}}_{\left[0.5:0.95\right]}$ results of min:0.49, max:0.812. We hope these datasets are useful and serve as a baseline for future studies.

## 1. Introduction

The recent advances in data-driven machine learning (ML) techniques have been unlocked and achieved impressive outcomes in industrial research sectors and even in our daily life. As exemplified by applications such as autonomous driving [1], natural language processing (NLP) [2], synthetic visual data generation [3], protein structure prediction [4], and nuclear fusion reactor control [5], it is very exciting to see what else ML can learn and how much it will bring impact to our future life.

**Figure 1:**
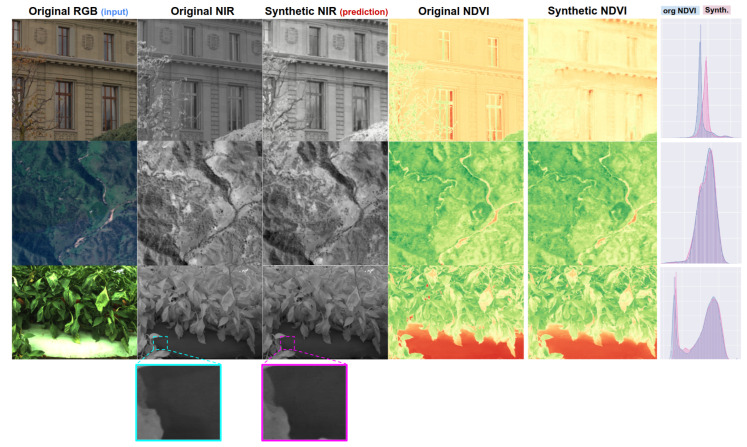
Each row indicates a data sample and the corresponding output drawn from nirscene, SEN12MS, and capsicum datasets respectively. The 1st and 2nd columns are RGB+NIR image pairs used for model training, 3rd is a NIR prediction given the RGB image and their normalised difference vegetation index (NDVI) for the rest of the columns. This figure is best viewed in colour.

In this paper, we are interested in bringing these data-driven ML technologies to the agriculture sector to take considerable advantages in core agricultural tasks such as vegetation segmentation and fruit detection. Toward this, we adopt one of the ML techniques, synthetic image generation and data-driven object detection, to see how much improvement we can obtain. Especially, we first focus on generating synthetic near-infrared (NIR) images that can then be used for object detection as auxiliary information, as shown in Figure 1.

Within the agriculture domain, NIR information (λ∼750–850 nm) has played a pivotal role in various tasks since 1970. One of the most important contributions is enabling vegetation indices (NDVI) with a simple and fast closed-form [6], and this still now sets a stepping stone for many other advanced indices such as the enhanced vegetation index (EVI) or normalized difference water index (NDWI). Analogous to the thermal spectrum, which allows measuring beyond the visible range and brings significant salient features, the NIR spectrum enables observing plants’ chlorophyll responses (mainly from leaves). This information is crucial for agronomists to phenotype vegetation’s status and conditions.

In order to synthesise NIR information from RGB input, it is necessary to properly approximate a highly nonlinear mapping, $f_{\theta }$ such that $f_{\theta }:\left\{I_{RGB}\right\}\mapsto I_{NIR}$ where θ is unknown parameters (e.g., neural networks’ parameters). The nonlinearity stems from incident lighting sources, surface reflectances, intrinsic and extrinsic camera inherent characteristics and many other factors. Hence, it is one of the challenges to estimate the global optimal solution that guarantees convergence. Instead, we attempt to learn the mapping in a data-driven, unsupervised manner that does not require manual annotations or labels. To do this, we set the objective function that minimises differences between synthetic and original NIR images given RGB images. This idea is straightforward, and there are already several previous studies in generating not only NIR [7,8] but also thermal [9] and depth [10,11].

This paper is different to [7,8] in the following aspects. First and most importantly, we clearly present experimental results with a high level of technical detail which is lacking in both. In synthetic image generation and generally machine learning, it is one of the important tasks to carefully split train and test sets to hold equivalent or similar statistical properties (e.g., feature distributions) among sets. However, none of them correctly disclosed this, and only present scores which are somehow meaningless. None of them made the dataset available for public use so it is impossible to reproduce or build another system on top of their studies. Lastly, it is vague why and how synthetically generated information is useful in their work. We demonstrate this by feeding forward synthetic NIR images into a subsequent fruit detection task. Therefore our contributions in this paper are:

- We made publicly available NIR+RGB datasets for synthetic image generation [12]. Note that we utilised 2 publicly available datasets, nirscene [13] and SEN12MS [14], in generating these synthetic datasets. For image pre-processing, oversampling with hard random cropping was applied to nirscene and image standardisation (i.e., μ=0, σ=1 was applied. It is the first time to realise the capsicum NIR+RGB dataset. All datasets are split in a 8:1:1 (train/validation/test) ratio. This dataset follows a standard format so that it is straightforward to be exploited with any other synthetic image generating engine.
- We expanded our previous study [15], and we added four more fruit categories including their bounding box annotations. A total of 11 fruit/crops were rigorously evaluated and to our best knowledge, this is the largest type of dataset currently available.

**Figure 2:**
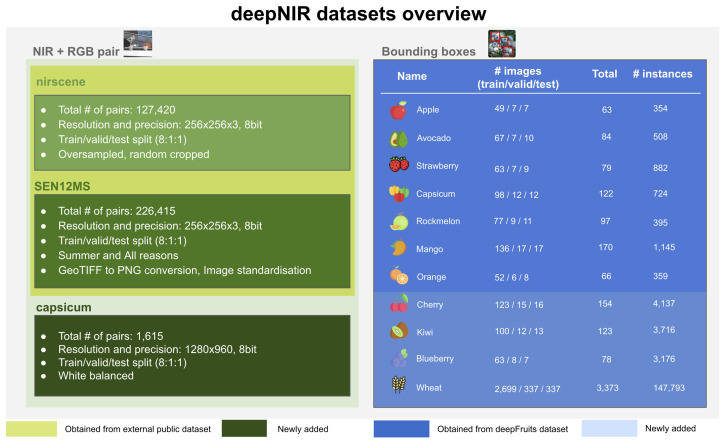
NIR+RGB pair and object detection datasets overview.

Figure 2 summarises all datasets presented in this paper. The left green boxes indicate NIR + RGB pair datasets and their technical detail and the right blue box denotes the bounding box dataset for object detection.

Other than the above contributions, we also present detailed experimental results, their analysis and insights that can be useful for readers. The dataset can be downloaded from http://tiny.one/deepNIR (accessed on 17 June 2022).

The rest of the paper is structured as follows. Section 2 presents literature reviews on synthetic image generation and object detection. Section 3 covers methodologies used for generating synthetic images and dataset detail and the concise summary of the generative adversarial network and a single-stage detection framework. Section 4 contains evaluation metrics for both synthetic image generation and object detection followed by qualitative and quantitative results. This also includes inter-comparisons between models developed in this paper and baseline comparisons with evaluations from other studies. We also discuss the advantages and limitations that we found in Section 5. Section 6 concludes the paper by giving a summary of results, impact of the proposed work and future outlook.

## 2. Related Work

In this section, we describe previous related studies; especially focusing on public RGB–NIR datasets, synthetic image generation and object detection approaches. This section presents a clear distinction between other datasets and what we proposed and state-of-the-art synthetic image generation and object detection techniques that other researchers can alternatively exploit. Therefore this section is helpful for readers to better understand the remaining sections.

### 2.1. Near-Infrared (NIR) and RGB Image Dataset

Modern data-driven deep learning approaches have demonstrated very promising and impressive performance in a variety of sectors, and it is no exaggeration to say that large-scale and high-quality training datasets have played a pivotal role in achieving these successes. Especially within the agricultural domain, NIR-RGB (or multispectral interchangeably) datasets provide rich feature information about vegetation such as crops or fruits. They are regarded as one of the important key indices for agronomists, data scientists and machine learning researchers. Hence there exist valuable and noticeable contributions [16,17,18,19,20] in broad-arcs, horticulture [21] or protected farm scenarios [22,23].

Brown et al. [13] is one of the frontier NIR–RGB datasets focusing on scene recognition tasks by utilising the multispectral information. As mentioned earlier, this dataset contains 477 RGB+NIR image pairs that were asynchronously captured using two cameras in mostly outdoor daily-life scenes. This dataset is useful, and we also utilised it in this paper. Still, a temporal discrepancy in a pair and a lack of radiometric calibration and small-scale datasets are challenges to the use of this dataset. We demonstrate the impact of dataset scale and oversampling strategy in the following section.

More recently, one of our previous studies [19,24] that focused on sweet pepper detection and semantic segmentation using multispectral images contributed to the horticulture sector. A total of 103 pixel-level annotations and NIR+RGB pairs were used in this work. Considering challenges in the agricultural scene pixel-labelling task, it was one of the novel datasets together with [25] that contributed 60 annotations even though the scale was relatively smaller than current datasets. More importantly, we share in this paper another 1615 NIR–RGB pair dataset collected in that campaign yet annotated. The summary of NIR-RGB dataset is presented in Table 1.

Chebrolu et al. [16] offered a comprehensive large-scale agricultural robot dataset that is suitable for vegetation semantic segmentation as well as localization and mapping. Multispectral images, RGB-D, LiDAR, RTK-GPS and wheel odometry sensor data were collected over a sugar beet field for two months in Germany. A total of 5 TB of the dataset was obtained, but [16] does not provide a dataset summary table, so it is rather difficult to find how many multispectral images and their annotations without attempting to use the dataset.

In agriculture, satellite imagery is one of the important resources that is widely utilised in many applications as a key source of information. They provide abundant, large-scale earth observations, which are useful for data-driven machine learning approaches. Therefore, promising studies [26] and a public dataset [14] utilised satellite multispectral imagery (e.g., Sentinel-2 A+B twin satellites platforms launched by the European Space Agency (ESA)).

Schmitt et al. [14] introduced an unprecedented multi-spectral dataset in 2019. They sampled from 256 globally distributed locations over four seasons which constitutes about 180 k NIR+RGB pairs. We adopt this dataset with the following processes in this paper. We converted the raw dataset formatted as multi-channel GeoTIFF into an ordinary image formation with image standardisation for each image pair and split it to train/validation/test sets. We agree that these steps are trivial and straightforward to achieve. However, in practice especially training a model using 180 k multispectral images, it often matters to know concise and exact split sets and to secure a direct trainable dataset rather than an ambiguous and questionable split [7,8] or requiring any modifications from a reproducibility perspective. This ambiguity can result in spending time and effort building up a baseline. For instance, converting a digital number (DN) to 8-bit per channel standard image format can be a non-trivial task, and evaluation metrics can also vary depending on how the dataset is split. From these perspectives, it is important to establish a fixed dataset split with the corresponding metric to see how much other factors impact the performance (i.e., ablation studies).

### 2.2. Synthetic Image Generation

Synthetic image generation is perhaps one of the most attractive and active fields among many other interesting and promising applications of deep learning techniques. Rooting from generative adversarial networks (GAN) [27], there are brilliant ideas that either improved the original adversarial idea or established another stepping stone.

Mirza et al. [28] proposed a new idea that conditionally takes not only noise input for a generator but auxiliary information for better model convergence and generalisation (cGAN). They demonstrated the impact for the image-to-image translation task and enabled/highly influenced other variants such as Pix2pix [29,30], StyleGAN [31], CycleGAN [32] or more recently OASIS [33]. Even though they propose different approaches and applications, the fundamental idea stems from the studies mentioned above. In this paper, we adopt the work of [30] in order to evaluate and confirm the implications of our dataset, but readers can freely choose any state-of-the-art framework as a synthetic generation tool.

On top of these core works, many interesting applications use synthetic image generation. Transforming from visible range (RGB) to near-infrared spectrum (NIR) is demonstrated in [7], and Ref. [8] using nirscene and SEN12MS datasets respectively. Aslahishahri et al. [34] showed aerial crop monitoring with synthetic NIR generation using a software package from [29]. Although they disclosed their dataset, its scale (only 12 pairs) is too marginal to use for model training effectively.

There were interesting studies that transformed from thermal range to visual spectrum [9,35]. The objective was the estimation of nonlinear mapping between visible spectrum (450∼750 nm) to long wave infrared (8∼12 μm) range. The large gap in the spectrum causes severe appearance differences, hence the task is more difficult than the NIR–RGB mapping case. In order to achieve a good generalised model and stable performance, a large-scale dataset and precise thermal calibration (e.g., fluid field correction and temperature calibration) are required.

Instead of separately treating each image, there were attempts to fuse only distinct features. The studies of [36,37] proposed a fusion approach of multimodal data and the goal of their work was to fuse visible texture from an RGB image and thermal radiation from an infrared image by forcing a discriminator to have more details.

As we can see from the above literature, it is key to choose proximal spectrum ranges to learn a nonlinear mapping successfully. Ma et al. [38] exemplified this by demonstrating transformation from NIR-I (900∼1300 nm) to NIR-IIb (1500∼1700 nm) in vivo fluorescence (an imaging technique applying glow substances to cells to record responses of live organisms). According to their results, they achieved unprecedented signal-to-background ratio and light-sheet microscopy resolution. A similar approach was applied to medical imagery [39]: generating magnetic resonance images (MRI) from computed tomography image (CTI) using CycleGAN [32] and unsupervised image-to-image translation network (UNIT) [40].

We have introduced the most fundamental studies and outstanding applications in our perspectives on image-to-image translation using GAN techniques. However this research field is active and developing at a fast pace, so we would like to refer to a more solid and recent survey paper [41,42].

### 2.3. Object-Based Fruit Localisation

Synthetically generated images from the previous section can be used as auxiliary information for various computer vision tasks such as object classification, recognition, bounding box-level detection, and semantic segmentation in order to improve performance. In this paper, we are interested in the fruit object detection (i.e., bounding box localisation) task following our previous studies presented in deepFruits [15] where we demonstrated seven fruits/crops detection using a two-stage object detector [43]. On top of the work, we share four additional novel fruit annotations and their split. We evaluated our 3- and 4-channel datasets using a single-stage detector [44].

The object detection problem is one of the most important tasks in remote sensing, computer vision, machine learning and robotics communities. Recent advances in large-scale datasets and machine learning algorithms accelerated by GPU computing have unlocked potential and achieved super human-level performance. In this research area, there are two main streams: single and two-stage detection.

The first is to formulate the problem as a single regression optimisation problem gaining faster inference speed with the cost of inferior performance [44,45]. Whereas the latter, two-stage detector employs a region proposal network (RPN), which suggests a number of candidates (e.g., rectangles or circle [46] primitives) for the subsequent object classification and bounding-box regression heads [43,47,48]. Generally, this approach achieved superior performance at the cost of processing speed. According to the recent trends in object detection, it is worth mentioning that the detection performance gap between these paradigms has been significantly reduced with the aid of intensive optimised image augmentation techniques and more efficient network architecture design [49]. We will discuss this more in Section 3.2.

Even though we presented the most remarkable achievements in the area, we would like to point out another object detection survey paper [50] that covers more concrete summaries and research directions.

## 3. Methodologies

In this section, we present synthetic near-infrared image generation and its application, object detection, by using the generated 4 channels (i.e., 3 × visible + 1 × infrared spectra) of data.

### 3.1. Synthetic Near-Infrared Image Generation

Abundant and high-quality training data is one of the essential driving factors, especially for data-driven approaches such as deep neural networks (DNN). Securing such data often requires a lot of resources (e.g., manual annotations). Therefore, researchers and communities have devoted tremendous efforts to this, which leads to impressive and brilliant ideas such as data augmentation [51,52], pseudo labelling [53], and generative adversarial models [29,30,33]. In this paper, we are interested in exploiting a generative model for the following reasons. First, it is straightforward to re-formulate the problem by adopting ideas from previous studies such as style-transfer [29] and fake image generation [54]. For the training phase, we only need to feed image pairs (RGB, NIR) as input and target. Second, there exist well-established resources that demonstrate outstanding performance in non-agricultural domains such as fake face image generation or style transfer from hand drawings to masterpieces.

**Figure 3:**
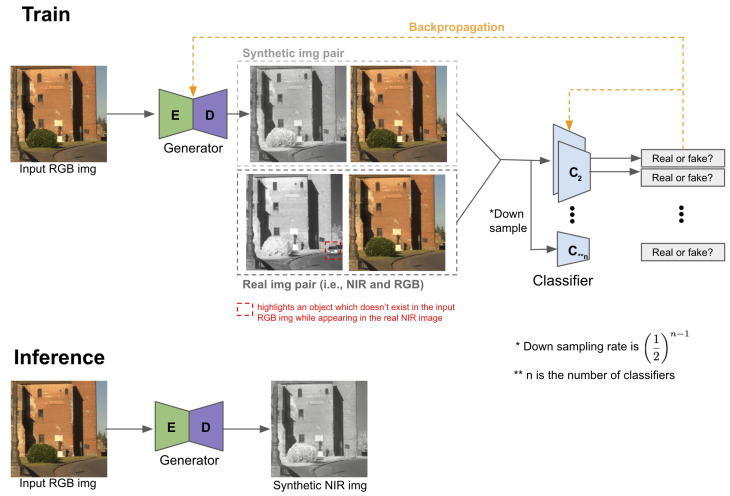
Synthetic image train (**top**) and inference (**bottom**) pipeline. This is one of typical type of conditional generative adversarial network (cGAN) [29]. Image redrawn from [30].

Figure 3 illustrates one of the generative adversarial networks (GAN) for synthetic image generation [30]. Our goal is to find the optimal generator and classifier given real image pairs at the training phase. As shown in the figure, the role of the generator and classifier is to create a synthetic image pair and distinguish real or synthetic pairs, respectively. The inference stage simply performs forward prediction using the trained generator model and input RGB image, creating synthetic image output. It is worth mentioning that the generator may have abilities to generate small obscured scenes if train and test datasets share a similar context. For instance, there is a passing car in the real NIR image but not in the RGB image in Figure 3. This happened because NIR and RGB images were asynchronously captured in the public dataset [13]. Despite this, our generator is able to recover the small portion of the image (red dashed box) because it learned how to transfer from RGB spectrum (380–740 nm) to NIR (∼750 nm).

More formally, the objective of GAN (conditional GAN [28] more precisely) can be expressed as:

(1) $$L_{\mathrm{GAN}}(G_{\theta_{G}},D_{\theta_{D}})=E_{x∼p_{data}\left(x\right),y}\left[logD_{\theta_{D}}\left(x|y\right)\right]+E_{z∼p_{z}\left(z\right)}[log\left(1-D_{\theta_{D}}\left(G_{\theta_{G}}\left(z|y\right))\right)\right]$$

where $G_{\theta_{G}}$ and $D_{\theta_{D}}$ are generator, G:{x,z}↦y and classifier (or discriminator) parameterised $\theta_{G}$ and $\theta_{D}$, respectively. $x\in R^{\mathrm{W\times H\times C}}$, $y\in R^{\mathrm{W\times H\times C}}$ and $z\in R^{1}$ are input image, target image and a random noise in this case. Intuitively the first term indicates the expectation of classifier given data sample *x* (i.e., RGB image) drawn from input data distribution and target *y*, which is an NIR image. Maximising this term implies we successfully fool the classifier even though the generator produces synthetic images. The second term is what we want to minimise the difference between the output of generator ($\in R^{\mathrm{W\times H\times C}}$) given random noise *z* drawn from noise distribution, $p_{z}\left(z\right)$, given target image *y* and target image *y* as close as possible.

Concretely we can also add the L1 loss function in order to minimise blurring

(2) $$L_{L1}\left(G_{\theta_{G}}\right)=E_{x,y,z}[||z-G_{\theta_{G}}\left(x|y\right){\left|\right|}_{1}]$$

Therefore, the final objective is a min-max optimisation problem

(3) $$arg\min_{G_{\theta_{G}}}\max_{D_{\theta_{D}}}L_{GAN}(G_{\theta_{G}},D_{\theta_{D})}+\lambda L_{L1}\left(G_{\theta_{G}}\right).$$

Other than conditional GAN, there are many GAN variants in formulating loss functions such as convolutional GAN [55] or cycle GAN [32] which also can be utilised for synthetic image generation tasks.

Among many possible approaches, we selected the Pix2pixHD framework [30] as our baseline study for the following reasons. It has been widely used among synthetic data generation tasks, so there are many comparable resources. It can handle higher resolution images than its ancestor [29] and is easy to use with many available options such as hyperparameter searching and model evaluation. We made datasets used in this paper for training and testing available for the public. One can reproduce or evaluate model performance using different state-of-the-art GAN frameworks.

#### Datasets Used for Generating Synthetic Image

We made minor modifications in the use of our baseline synthetic image generation framework (i.e., Pix2pixHD) to be able to evaluate model performance while varying datasets, as shown in Table 2. With these datasets, one will be able to reproduce similar results to those we achieved or use other frameworks for superior outcomes.

**Figure 4:**
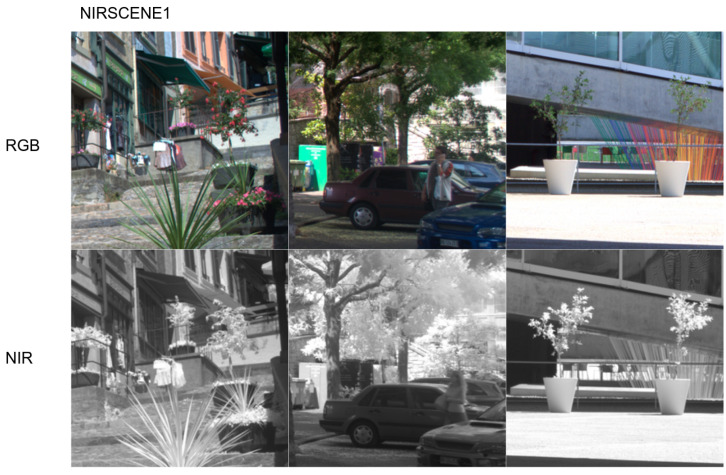
RGB (**top row**) and NIR (**bottom row**) samples drawn from our nirscene1 dataset (256 × 256). This dataset was mostly taken in outdoor environments with band-cutoff filter equipped two digital single-lens reflex (DSLR) cameras. Note that there also exists noticeable temporal gap between NIR and RGB images (**middle column**).

The data consist of three public [13,14,19] datasets. The first dataset namely, *nirscene1*, contains 477 RGB+NIR images (1024 × 679) that were captured with commercial high-end cameras with a 750 nm band-cutoff filter. The colour images were white-balanced, and the channel-wise average was applied to the infrared images. Two image alignment (or registration) was done through feature matching in RGB and NIR domains. These are only critical characteristics, but more technical detail can be found from [13]. Figure 4 shows sample images from the dataset. This dataset is useful but only has 477 pairs which may hinder a good visible-infrared domain mapping. Although more experimental results will be presented in the following Section 4, we performed hard-cropping and over-sampling to resolve this issue. Hard-cropping is one of the augmentation techniques which generates cropped datasets, whereas soft-cropping generates cropped samples during the training/testing phase. Over-sampling refers to randomly sampling more redundant data. It is a fact that the maximum amount of information we can get from the over-sampling is the original data. However, we found that over-sampling helped stabilise training and improved performance by a large margin. Regarding this, we will discuss and analyse more in Section 4.

**Figure 5:**
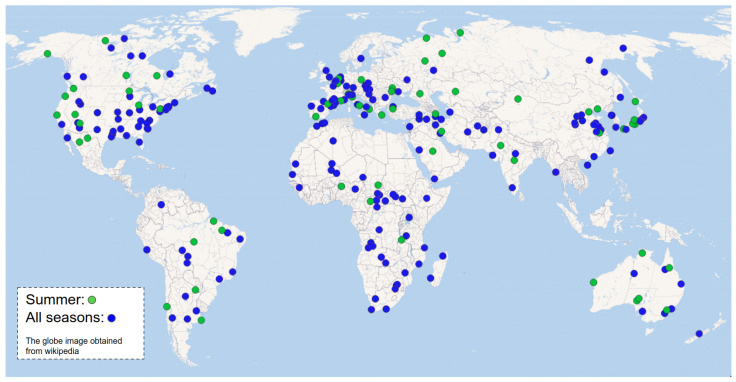
Green indicates 65 sampled locations from the SEN12MS Summer dataset and blue covers All seasons and 256 globally distributed locations in total.

**Figure 6:**
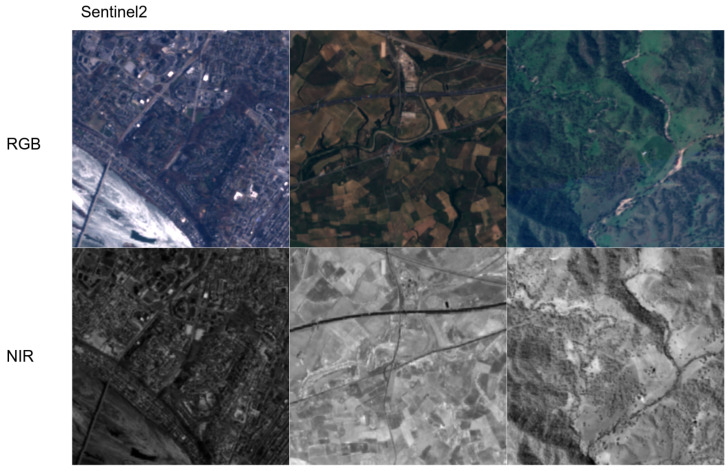
RGB (**top row**) and NIR (**bottom row**) samples (each column) from our SEN12MS dataset (random samples in winter and spring). Note that left RGB image’s brightness is adjusted for better visualisation. The original RGB image is darker.

The second dataset, *SEN12MS*, is publicly available satellite imagery from Sentinel-1 and Sentinel-2. No image processing is applied to this dataset, we only selected two subsets (Summer and All seasons) followed by a train/valid/test split. Figure 5 shows geolocations where the authors sampled multispectral imagery. A multispectral image covers spectral range from 450–842 nm (i.e., band2, band3, band4, band8 of Sentinel-2) and captured at 768 km. This leads to having 10 m ground sample distance (GSD)/pixel. Radiometric calibration was properly performed by the satellite system organisation. Figure 6 demonstrates a couple of samples in this dataset.

**Figure 7:**
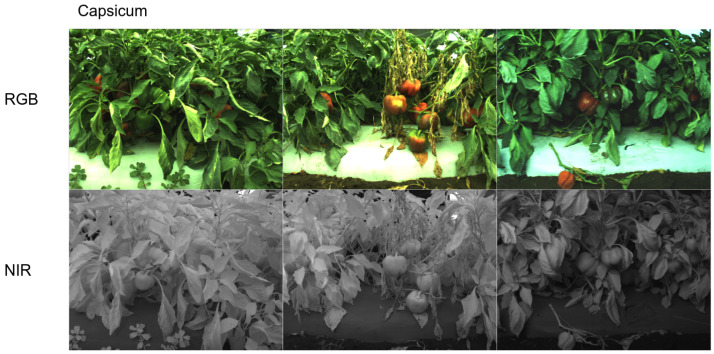
RGB (**top row**) and NIR (**bottom row**) samples (each column) from our capsicum dataset. It is one of the most challenging datasets, containing very cluttered and complex scenes in close proximity. Note that right RGB image’s brightness is adjusted for better visualisation. The original RGB image is darker.

The last dataset, *capsicum*, is one of our previous studies presented in [15,19]. The dataset was collected from sweet pepper farms in Australia, Gatton and Stanthorpe, with a multispectral camera, JAI AD-130GE. This camera has two charges coupled device (1280 × 960) prism mechanisms for each RGB and NIR spectrum. Unlike other datasets, we use the larger original image to train our model because this simplifies the subsequent procedures (e.g., object detection). Data collection campaigns were mostly performed at night with controlled visible and infrared light sources to mitigate external interference. White balance was properly performed with a grey chart, and radiometric calibration was omitted. Figure 7 shows samples from this dataset.

### 3.2. Fruit Detection Using Synthetic Images

In this section, we present one of the applications for which we can take advantage of synthetically generated images from the previous step. Object detection or semantic image segmentation is one of the important downstream tasks in many research and commercial areas. Especially, precise object detection in agriculture can be considered a pivotal stepping stone because it can be used for many other subsequent tasks such as crop counting, yield estimation, harvesting and disease detection with bounding-box level segmentation or its classification.

For objection detection, we chose Yolov5 [44] mainly due to its fast inference time (i.e., single-stage detection), the fact that it is easy to train and its intuitive visualisation advantages over other frameworks. However, there exist other powerful frameworks such SAHI [56], Detectron2 [57] or MMDetection [49] that can also be exploited. These frameworks are very flexible in adapting new modules or datasets and support many pretrained weights which can improve object detection performance by a large margin.

The network architecture and implementation details of Yolov5 can be found from [44], and we present only a concise high-level view in order to help readers in understanding object detection.

It consists of four sub-parts, namely input, backbone, neck and head layers. The first input layer adopts mosaic data augmentation that is an aggregation of cropped images, adaptive anchor, and many other augmentation techniques [51]. Backbone and neck networks are in charge of feature extraction by making use of focus (i.e., image slicing), convolution-batch-normalisation, and leaky ReLU (CBL), cross-stage-partial (CSP) [58] and spatial pyramid pooling (SPP) [59], feature pyramid networks (FPN) [60] and path aggregation network (PAN) [61] modules. Intuitively, the output of the neck network is feature pyramids that incorporate varying object scales, which may lead to superior performance than other single-stage-detectors (SSD). The last head layer is an application-specific layer, and most object detection tasks predict bounding box (4), confidence (4), and class (1) from the head layer. More concretely, the bounding box loss $L_{box}$ used in the object detection is expressed

(4) $$\begin{array}{cc} L_{\mathrm{box}}= & \sum_{i=0}^{s\times s}\sum_{j=0}^{N}I_{i,j}^{\mathrm{obj}}\left(1-\mathrm{GIoU}\right) \end{array}$$

(5) $$\begin{array}{cc} I_{i,j}^{\mathrm{obj}}= & \left\{\begin{array}{cc} 1 & \mathrm{if}\;\mathrm{prediction}\;\mathrm{exists}\;\mathrm{within}\;\mathrm{annotation}\\ 0 & \mathrm{otherwise}. \end{array}\right. \end{array}$$

(6) $$\begin{array}{cc} \mathrm{where}\;\mathrm{GIoU}= & \mathrm{IoU}-\frac{C-U}{C} \end{array}$$

(7) $$\begin{array}{cc} \mathrm{IoU}= & \frac{I}{U},\;\;\;U=\hat{B}+B-I \end{array}$$

where *s* is the number of a grid, *N* is the number of bounding boxes in each grid. GIoU is generalised intersection over union [62] which has [−1, 1] scalar value. $\hat{B}$, *B* are area of prediction and annotation bounding boxes (i.e., two arbitrary convex shapes) and *C* is the area of the smallest enclosing convex shape. I and U are the intersection and union of $\hat{B}$ and *B* respectively. IoU indicates intersection over union of $\hat{B}$ and *B*. Intuition of the loss function is that the loss will keep increasing with smaller GIoU implying smaller overlap between $\hat{B}$ and *B*, on the other hand the loss decreases with larger GIoU when two bounding boxes are largely overlapped.

There are two more losses for confidence score ($L_{\mathrm{score}}$) and class probability ($L_{\mathrm{class}}$) and they are modelled by logistic regression and binary cross entropy as follows:

$$\begin{array}{c} L_{\mathrm{score}}=-\sum_{i=0}^{s\times s}\sum_{j=0}^{N}I_{i,j}^{\mathrm{obj}}\left(S_{i}^{j}\log\left({\hat{S}}_{i}^{j}\right)+\left(1-S_{i}^{j}\right)\log\left(1-{\hat{S}}_{i}^{j}\right)\right)\\ \;\;-\lambda_{\mathrm{empty}}\sum_{i=0}^{s\times s}\sum_{j=0}^{N}I_{i,j}^{\mathrm{empty}}\left(S_{i}^{j}\log\left({\hat{S}}_{i}^{j}\right)+\left(1-S_{i}^{j}\right)\log\left(1-{\hat{S}}_{i}^{j}\right)\right)\\ I_{i,j}^{\mathrm{empty}}=\left\{\begin{array}{cc} 1 & \mathrm{if}\;\mathrm{there}\;\mathrm{is}\;\mathrm{no}\;\mathrm{prediction}\\ 0 & \mathrm{otherwise}. \end{array}\right. \end{array}$$

where ${\hat{S}}_{i}^{j}$ and $S_{i}^{j}$ indicate the prediction and annotation scores (usually = 1.0) of the *j*-th bounding box in the *i*-th grid. $\lambda_{\mathrm{empty}}$ is the weight when there is no predicted object within the *j*-th bounding box.

Similarly, class probability $L_{\mathrm{cls}}$ is defined as

(8) $$\begin{array}{c} L_{\mathrm{cls}}=-\sum_{i=0}^{s\times s}\sum_{j=0}^{N}I_{i,j}^{\mathrm{obj}}\left(P_{i}^{j}\log\left({\hat{P}}_{i}^{j}\right)+\left(1-P_{i}^{j}\right)\log\left(1-{\hat{P}}_{i}^{j}\right)\right) \end{array}$$

(9) $$\begin{array}{c} \;\;-\lambda_{\mathrm{empty}}\sum_{i=0}^{s\times s}\sum_{j=0}^{N}I_{i,j}^{\mathrm{empty}}\left(P_{i}^{j}\log\left({\hat{P}}_{i}^{j}\right)+\left(1-P_{i}^{j}\right)\log\left(1-{\hat{P}}_{i}^{j}\right)\right) \end{array}$$

The total loss can be calculated

(10) $$\begin{array}{c} L_{\mathrm{total}}=L_{\mathrm{box}}+L_{\mathrm{score}}+L_{\mathrm{cls}} \end{array}$$

and we seek parameters that minimise the total loss in the training phase.

**Figure 8:**
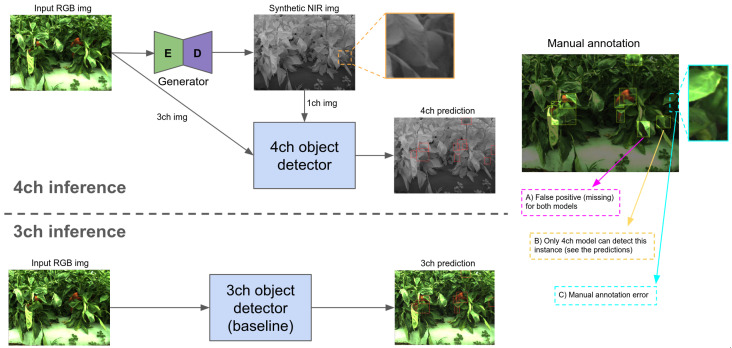
The 4-channel(ch) early fusion object detection inference pipeline (**top**) and 3-channel ordinary inference (**bottom**). Brighter areas in manual annotation on the right image indicate manual labeled bounding boxes. One can compare them with model predictions (i.e., red boxes in ‘4ch prediction’ and ‘3ch prediction’). This image best viewed zoomed-in.

Figure 8 illustrates the object detection pipeline we proposed in this paper. Among various visual fusion approaches, we follow ‘early fusion’ in order to maintain a similar inference processing time as of 3 channel inference (‘late fusion’ requires O(N) complexity where *N* is the number of input). Moreover, it is easier to implement and straightforward to extend from 3 channel baseline. Firstly, input image $\in R^{W\times H\times 3}$ is fed into the generator that learnt mapping from visible-to-infrared domain and outputs a synthetic image $\in R^{W\times H\times 1}$. These two data are concatenated to form the shape of input data $\in R^{W\times H\times 4}$ prior to the input convolution layer (i.e., early fusion). After forward computation, the network predicts bounding boxes with the corresponding confidence (see red boxes in the figure). Here ‘4ch inference’ and ‘3ch inference’ indicate this prediction with synthetic image and without it, respectively. From this cherry-picked experiment, we observed interesting aspects: (1) there is an instance that only the 4-channel model can detect. Marked in yellow from manual annotation, the 3-channel model missed (false negative) capsicum obscured by leaves and severe shadow. In comparison, the 4-channel model correctly detected it, and we believe this is the impact of introducing synthetic images; (2) Both models failed in very challenging instances (marked in magenta); (3) Both models successfully detect the object despite manual annotation error (marked in cyan).

#### Datasets Used for Fruit Detection (4ch)

In order to see the impact of synthetically generated images, we created a dataset that includes 4 channels and **11** fruits built upon our previous study [15] which presented 7 fruit for detection as shown in Table 3. Blueberry, cherry, kiwi, and wheat are newly introduced in this dataset. Even though the total number of images is far less than other publicly available datasets such as ImageNet and COCO (except wheat), this may be useful for mode pre-training for another downstream task. Each image contains multiple instances because fruits usually form a cluster. In addition to this, each fruit image was taken in various camera views, scale and lighting conditions which are very helpful for model generalisation. We made this dataset publicly available in a cloud annotation framework so that one can download them in many different formats (https://tiny.one/deepNIR (accessed on 17 June 2022)). Note that we manually generated and fixed errors in our previous dataset [15] except the wheat dataset obtained from a machine learning competition (https://www.kaggle.com/c/global-wheat-detection (accessed on 17 June 2022)). The dataset split followed the 8:1:1 rule for train/validation/test and final object detection results were reported using the test set. Detailed experiment results and dataset samples are presented in the following experiments section.

## 4. Experiments and Results

In this section, we first define evaluation metrics used for synthetically generated images and object detection tasks. Based on these, quantitative and qualitative results are presented for both synthetic NIR image generation and object detection tasks.

### 4.1. Evaluation Metrics

For synthetic image generation, it is relatively difficult to accurately gauge performance because the generator model often produces imaginary images (e.g., fake faces). Fortunately, in our task, we can utilise either traditional image similarity metrics or feature space image distribution comparisons because our objective is to generate a synthetic NIR image with a small residual error compared to the original NIR. For the object detection task, we adopt mean average precision with IoU sweeping range from [0.5:0.95] (${\mathrm{mAP}}_{\left[0.5:0.95\right]}$) with a 0.05 step.

#### 4.1.1. Synthetic Image Evaluation Metrics

As mentioned with inherent challenges in evaluating synthetic images, communities widely have used various performance metrics [63] such as Frechet inception distance (FID) or generative adversarial metric (GAM). Each of them has its particular advantages and disadvantages. Among them, we choose FID which reports image similarity between two images in high-dimensional feature space. It implies the metric finds the distance between two multivariate Gaussian distributions, $X_{A}\;∼\;N\left(\mu_{A},\sum_{A}\right)$ and $X_{B}∼N\left(\mu_{B},\sum_{B}\right)$ that are fitted to data embedded into a feature space (e.g., extracted features using InceptionNet or VGG16 backbone).

(11) $$\begin{array}{c} d_{\mathrm{FID}}\left(A,B\right)=\left|\right|\mu_{A}-\mu_{B}{\left|\right|}_{2}^{2}+Tr\left(\sum_{A}+\sum_{B}-2\sqrt{\mu_{A}\mu_{B}}\right) \end{array}$$

#### 4.1.2. Object Detection Evaluation Metric

There are also many metrics for object detection tasks such as IoU, GIoU, mAP and F-α [64]. Among them, ${\mathrm{mAP}}_{\left[0.5:0.95\right]}$ is a widely utilised and acceptable metric [65] and it is defined as follows

(12) $$\begin{array}{cc} P= & \frac{TP}{TP+FP} \end{array}$$

(13) $$\begin{array}{cc} R= & \frac{TP}{TP+FN} \end{array}$$

(14) $$\begin{array}{cc} AP= & \int_{0}^{1}P\left(R\right)\delta R \end{array}$$

(15) $$\begin{array}{cc} mAP= & \frac{1}{M}\sum_{i=1}^{M}{\mathrm{AP}}_{i} \end{array}$$

where TP, FP and FN denote true-positive, false-positive and false-negative, respectively. True positive implies our prediction is correct as of annotation bounding box (hit), false positive is when we wrongly make a prediction (false-alarm), and false-negative occurs when we miss a bounding box (miss). Note that true negative TN is not considered in object detection task because this implies correct rejection (e.g., there should not be an bounding box and a model does not predict at that location) and there exist infinite cases that satisfy the condition. *P* and *R* are precision and recall, and *AP* and *mAP* refers to average precision, and mean average precision that is the mean of all class’s *AP*. In our case, *AP* and *mAP* are treated equally because we only have one class (M=1) in our training dataset. As shown, *AP* is equal to the area of the precision–recall curve and ${\mathrm{mAP}}_{\left[0.5:0.95\right]}$ is the mean average precision of all classes while varying IoU threshold range from 0.5 to 0.95 with 0.05 steps (i.e., mean of total 20 samples).

In terms of train/inference processing time per dataset and GPU devices used for each task, Table 4.

### 4.2. Quantitative Results for Synthetic NIR Image Generation

In this section, we present three quantitative synthetic image generation results for nirscene1, SEN12MS and capsicum datasets.

An et al. demonstrated impressive results by making use of the multi-channel attention selection module [7]. They cropped 3691 images with 256 × 256 resolution for model training and testing. Unfortunately, the dataset used in this study is unavailable and technical details are insufficient for fair comparisons (e.g., train/test samples and their split is not disclosed).

As a rule of thumb, we split our dataset 8:1:1 for train/validation/test as described in Table 2 and proceeded with experiments. We achieved comparable results as shown in Table 5. It can be observed that FID keeps improving (lower is better), corresponding to oversampling rate. It is a fact that the maximum amount of information from the oversampled samples should be less than or equal to the original dataset. This redundancy may introduce system overhead as mentioned in [66] and make a marginal impact on image segmentation or object detection tasks. However, training the GAN-style model often demonstrates distinct characteristics. Speaking of convergence, it is often difficult to find the optimal point for GAN models because of its naturally inherent min-max game framework. Even with stable convergence, it is commonly difficult to guarantee the performance of the trained GAN model due to mode-collapse or diminished gradient issues [67]. If the number of samples is small (e.g., <10 k), it is very challenging to train a stable model. By oversampling, we hypothesise that GAN models can learn stable parameters significantly affected by the number of samples, especially batch-normalisation layers. This operation, namely hard-cropping, can also be done during the training phase by utilising augmentation approaches (soft-cropping). However, this soft cropping often is performed based on user-defined probability and may introduce more instability. The hard-cropping effect can be achieved with the maximum probability and a large number of epochs.

To our best knowledge, An et al. [7] is the only comparable baseline that exploited the nirscene1 dataset reporting FID score. However, this study inaccurately describes essential technical details such as how many test images were evaluated and data split. Therefore, it is difficult to make a fair comparison between our results and [7].

Yuan et al. [8] reported impressive results using the SEN12MS dataset for synthetic image generation. A total of 30,000/300 images were randomly sampled from the Summer dataset for training and testing, respectively. They reported quantitative results with image similarity metrics such as mean absolute error (MAE) or structural similarity (SSIM), which differ from our evaluation metrics. More importantly, the split strategy is questionable as only 1% was evaluated. The other 99% of the dataset was utilised for the training. From our perspective, with such small test samples, it is difficult to evaluate the model performance properly.

The capsicum data reported a 40.15 FID score. This dataset is the closest range among all datasets and contains cluttered structures and complex scenes. It is difficult for models to learn RGB to NIR mapping (or vice-versa) properly.

We summarise from these quantitative results that the number of samples is significant for the GAN model. Our synthetic NIR generator worked best for SEN12MS which performed proper radiometric calibration to have access to reflectances rather than raw pixel value. Accessing these reflectances is critical because they can hold consistent values despite acceptable changes in camera intrinsic or extrinsic parameters if images were taken under a similar light source. It is worth mentioning that image resolution is also one of the interesting aspects to consider, as shown in the higher-resolution result. If an image has a higher resolution, the more difficult it is for a network to learn the NIR–RGB relationship. In order to improve this, we are required to design a deeper network with more training samples.

The final analysis point is that the FID score is unitless and does not have a quality measure. A low score means high performance, but it is questionable whether 15 FID is good or bad. We suggest conducting a visual inspection of model prediction to address this issue, as presented in the following section.

### 4.3. Qualitative Results for Synthetic NIR Image Generation

**Figure 9:**
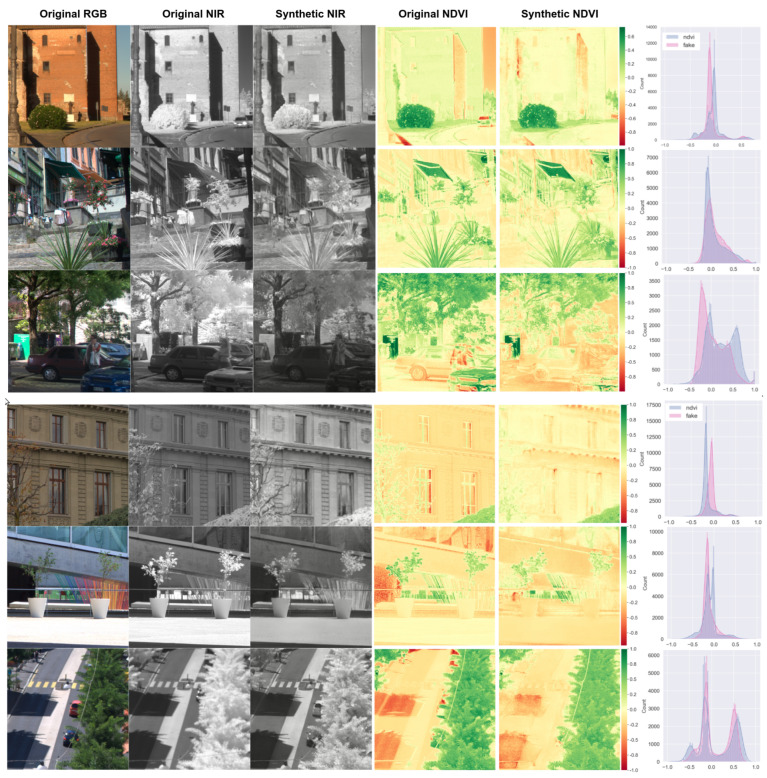
Six samples of nirscene1 dataset (1st and 2nd columns) and their corresponding synthetic NIR image (3rd) and NDVI images generated from original and synthetic NIR images (4th and 5th). The right most column shows pixel distribution for each NDVI images (red: synthetic, blue: original).

We show qualitative results of three datasets, nirscene1, SEN12MS and capsicum. The nirscene1 dataset has a FID of 26.53 and Figure 9 exemplifies six randomly selected test samples. The leftmost two columns are the original NIR–RGB pair and synthetic NIR refers to model prediction ($\hat{\mathrm{NIR}}$) that resulted in the mentioned FID score. Original NDVI indicates extracted NDVI $\frac{\mathrm{NIR}-\mathrm{RED}}{\mathrm{NIR}+\mathrm{RED}}$ using original NIR–RGB pair and synthetic NDVI is output by calculating $\frac{\hat{\mathrm{NIR}}-\mathrm{RED}}{\hat{\mathrm{NIR}}+\mathrm{RED}}$. These two NDVI images’ histograms are presented in the rightmost column (blue = original NDVI, red = synthetic). Generally speaking, the network learnt good nonlinear mapping. However, it shows limitations especially varying intensities and blurred edges. These are clearly noted from the histogram. The nirscene1 dataset contains many images taken outdoors where the light condition is inconsistent. This poses challenging conditions for the model to learn the mapping given a small dataset.

**Figure 10:**
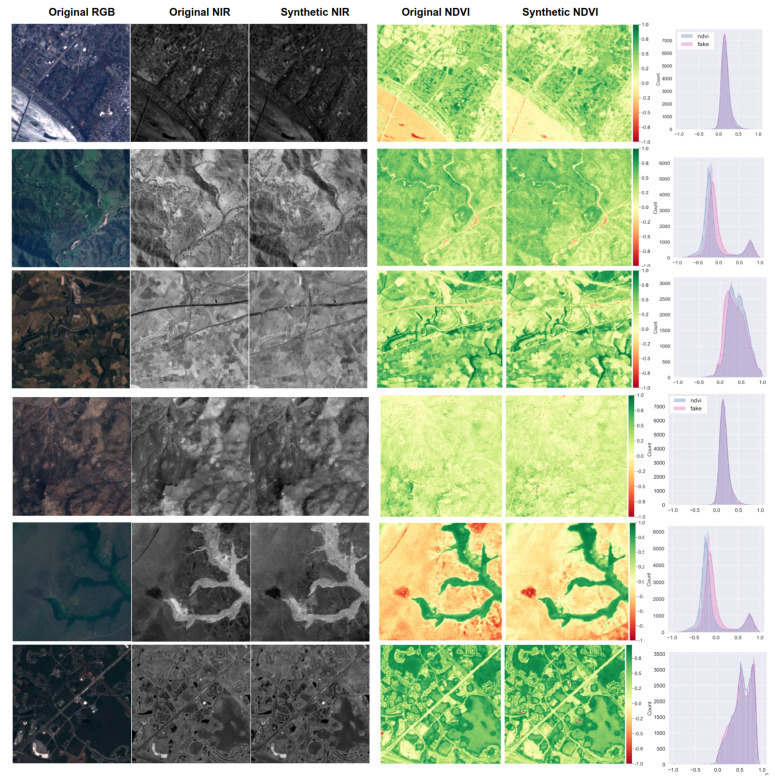
Six samples of SEN12MS dataset (1st and 2nd columns) and their corresponding synthetic NIR image (3rd) and NDVI images generated from original and synthetic NIR images (4th and 5th). The right most column shows pixel distribution for each NDVI images (red: synthetic, blue: original).

The SEN12MS dataset was taken/calibrated in more stable conditions than nirscene1. This clearly can be observed from Figure 10. Prediction nicely fits the original NDVI except for a couple of under-estimated points. We achieved an FID of 16.47 for the Summer and 11.36 for All season subsets in these experiments. Qualitatively speaking, it can be said that less than 15 FID is an excellent approximation of the original data. We are still investigating the reason for the underestimation. However, it is maybe a valid hypothesis that the network requires to see more images containing water as it mostly causes an error at very low NDVI, which is close to the normalised difference water index (NDWI) wavelength.

**Figure 11:**
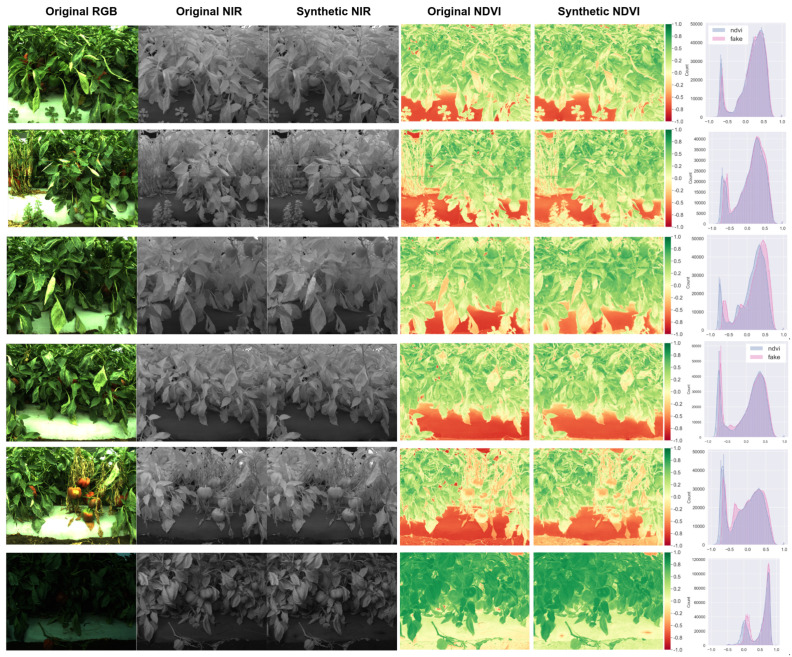
Six samples of capsicum dataset (1st and 2nd columns) and their corresponding synthetic NIR image (3rd) and NDVI images generated from original and synthetic NIR images (4th and 5th). The right most column shows pixel distribution for each NDVI images (red: synthetic, blue: original).

The last dataset is capsicum, as shown in Figure 11. This dataset holds consistent illumination and white balance, but some samples are severely under-exposed (see the bottom row in the figure). In addition, this dataset was taken in the closest range, causing very cluttered and complex scenes. Although our model worked surprisingly well, it still could not recover sharp detail that led to a relatively higher FID of 40.15, but it is still impressive, and we can use this result to see if we can improve the object detection task.

### 4.4. Applications for Fruit Detection Using Synthetic NIR and RGB Images

Synthetic NIR images (700–800 nm wavelength) can provide useful information that visible-range RGB images can not cover. One of the prominent properties is high reflectance in this particular bandwidth from vegetation due to chlorophyll in cells of leaves. Combining NIR with RED channel (i.e., normalised difference vegetation index (NDVI) [6]) enables the measurement of vegetated areas and their condition easily. Therefore, additional NIR information can significantly improve the performance of plant segmentation tasks as demonstrated in our previous studies [17,18].

Not only for the image segmentation task, but it can boost object detection performance as depicted in our previous experiments [15]. The provided information helps to enhance distinguishing power by providing quality features. For example, texture and objects under shallow shadow appear clearer in the infrared range than visible.

Thus, in this section, we aim at improving object detection performance by injecting additional synthetic data that we generated in the previous section. All experiments are conducted using the dataset mentioned in Table 3. We adopt the Yolov5 single-stage detector for the experiments and made minor modifications in order to take 4-channel input. Any other object detectors such as Detectron2, MMDetection or other Yolo-series can be easily utilised.

### 4.5. Quantitative Fruit Detection Results

As mentioned in Section 4.1, ${\mathrm{mAP}}_{\left[0.5:0.95\right]}$ is the key performance metric in this quantitative evaluation. A total of 11 fruits and crops are considered, as shown in Table 6. Note that seven fruits such as apple, avocado, capsicum, mango, orange rockmelon and strawberry were adopted from our previous work [15] when a two-stage detector (faster-RCNN [43]) was utilised as the main object detector. Although the samples for these seven fruits remain almost identical, we re-annotated wrongly annotated samples and made all of them available via cloud service with the four newly added fruit. This allows users to export the dataset in various formats suitable for seamlessly using various object detection frameworks with minimal effort.

We considered two models for each fruit, yolov5s (7.2 M parameters) and yolov5x (86.7 M), and each model is trained with/without synthetic NIR images. Therefore each fruit holds four performance results. All hyperparameters used are default from the latest repository, only the number of the epoch is set as 600 except for wheat, mainly due to being an order of magnitude larger in dataset size. Bolder indicates each fruit’s best score corresponding to the metric. During the training phase, we assume only one class exists in a dataset which is a valid assumption considering mono-species fruit farm scenarios. In generating synthetic NIR images, we deployed the generator trained with the capsicum dataset mentioned in Table 2. It is worth mentioning that the two capsicum datasets used for synthetic NIR generation and object detection are different in various aspects such as data collection campaign location, time and lighting condition. This is because we can only obtain capsicum annotations from our previous work [15] for object detection, whereas we have abundant un-annotated RGB+NIR pairs collected from trial collections for synthetic NIR generation.

Overall, all detection performances are impressive despite a small number of training samples. ${\mathrm{mAP}}_{0.5}$ shows a min-max of 0.85–0.98 and ${\mathrm{mAP}}_{\left[0.5:0.95\right]}$ of 0.49–0.81. Four fruits, apple, capsicum, avocado and orange, outperform by making use of additional NIR information, while seven others report the best performance only with RGB information. This result points against our objective, and we would like to elaborate on its causality deeply.

Stable and consistent reflectance plays a significantly important role in synthetic image generation. Intuitively, this implies our network is required to learn an RGB to NIR nonlinear mapping with small variations. If distributions and characteristics in the dataset significantly vary, our model would require more datasets covering the envelope. Otherwise, it will be under- or over-fitted, which in turn leads to inferior performance that occurred in our case. All datasets evaluated in object detection have a marginal correlation with the capsicum dataset utilised for GAN model learning. Many of them were obtained from web pages without NIR images. More detailed limitations, failure cases and possible workarounds are discussed in the next Section 5.

**Figure 12:**
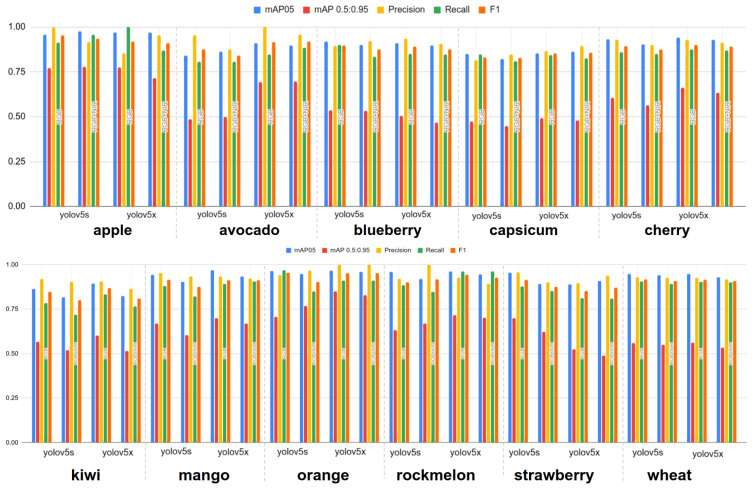
Object detection results summary. Different colours indicate the corresponding metrics. The different type of input data (i.e., RGB or RGB+NIR) are separately grouped for each yolov5 models.

Figure 12 shows a different view of Table 6. It is clear that a model with more parameters performs better at the cost of longer training time and hardware resources. At a first glance, the performance gap between only RGB and RGB+NIR is difficult to distinguish. To our best knowledge, it is sufficient to exploit only RGB images for fruits bounding box detection.

**Figure 13:**
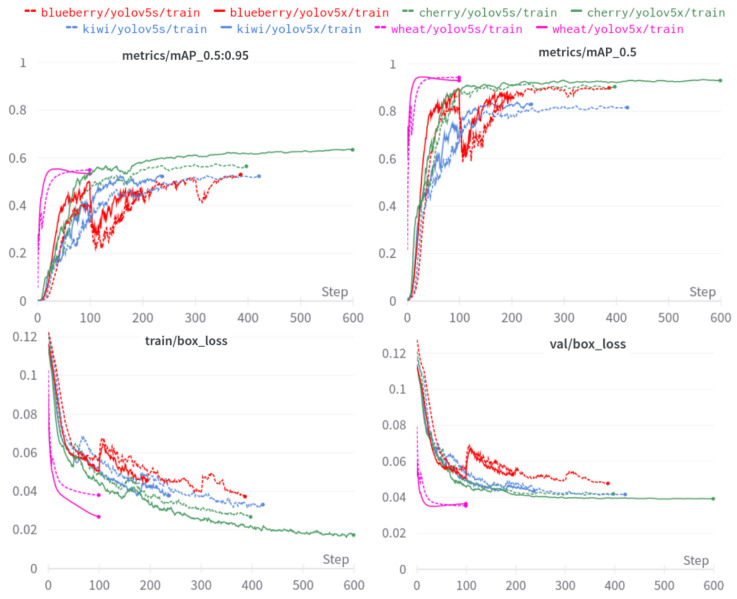
mAP performance metrics and train/validation bounding box loss plots for 4 newly added fruits/crops. Due to early-stopping mechanism, each experiment has a varying step length but it should have the same length for its metric and loss.

Train/validation losses are important measures to see the model’s performance and behaviours. Figure 13 reports two mAP metrics and losses results for newly added fruits, blueberry, cherry, kiwi and wheat. An early-stopping mechanism that terminates the training phase if the model’s evaluation has not improved for N=10 consecutive epoch/steps was activated, causing different steps for each fruit. All fruits are nicely converged without over-fitting and achieve impressive mAP. There are bumps around 100 steps for blueberry (red), and it is a common effect of image augmentation (e.g., probability-based geometry or colour transforms) while training.

### 4.6. Qualitative Object Detection Results

In this section, we demonstrate qualitative fruit detection performance. All images are randomly drawn from test sets (i.e., unseen data while training), and the best performing model, Yolov5x, with RGB images, is utilised for inference. In terms of inference time, it took 4.2 ms (238 Hz) and 10.3 ms (97 Hz) average inference time/image for Yolov5s and Yolov5x models given 640 × 640 resized image on NVIDIA RTX 3090 GPU, respectively. This inference time is matched with what Yolov5 reported [44] and is sufficient for real-time processing applications.

**Figure 14:**
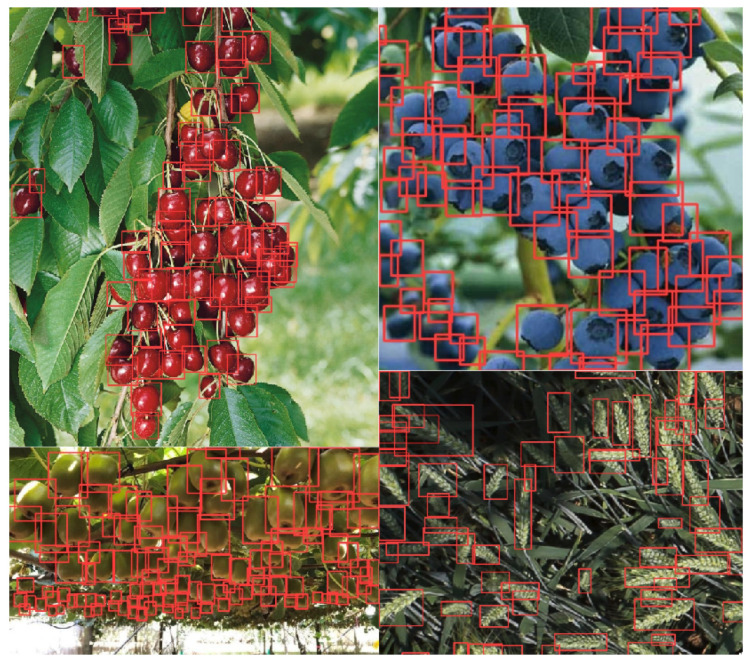
Four newly added fruits/crop prediction results using Yolov5x and RGB test images. Images are obtained from Google Images and Kaggle wheat detection competition (https://www.kaggle.com/c/global-wheat-detection) (accessed on 17 June 2022).

Despite the fact that we used the same training dataset of seven fruits as used in our previous study [15] in 2015, we can qualitatively observe performance improvement in the state-of-the-art object detector. This can clearly be seen from detecting small scale objects as depicted in Figure 14. Object detection has been actively developed since the early era of deep learning and achieved outstanding performance enhancement in accuracy and inference speed by developing strong image augmentations, model architectures, and hardware and software optimisation. Figure 14 exemplifies the detection results for 11 fruits. Due to length, we refer to the Supplementary Materials for all predictions of the 11 fruits presented in this paper.

## 5. Remaining Challenges and Limitations

**Figure 15:**
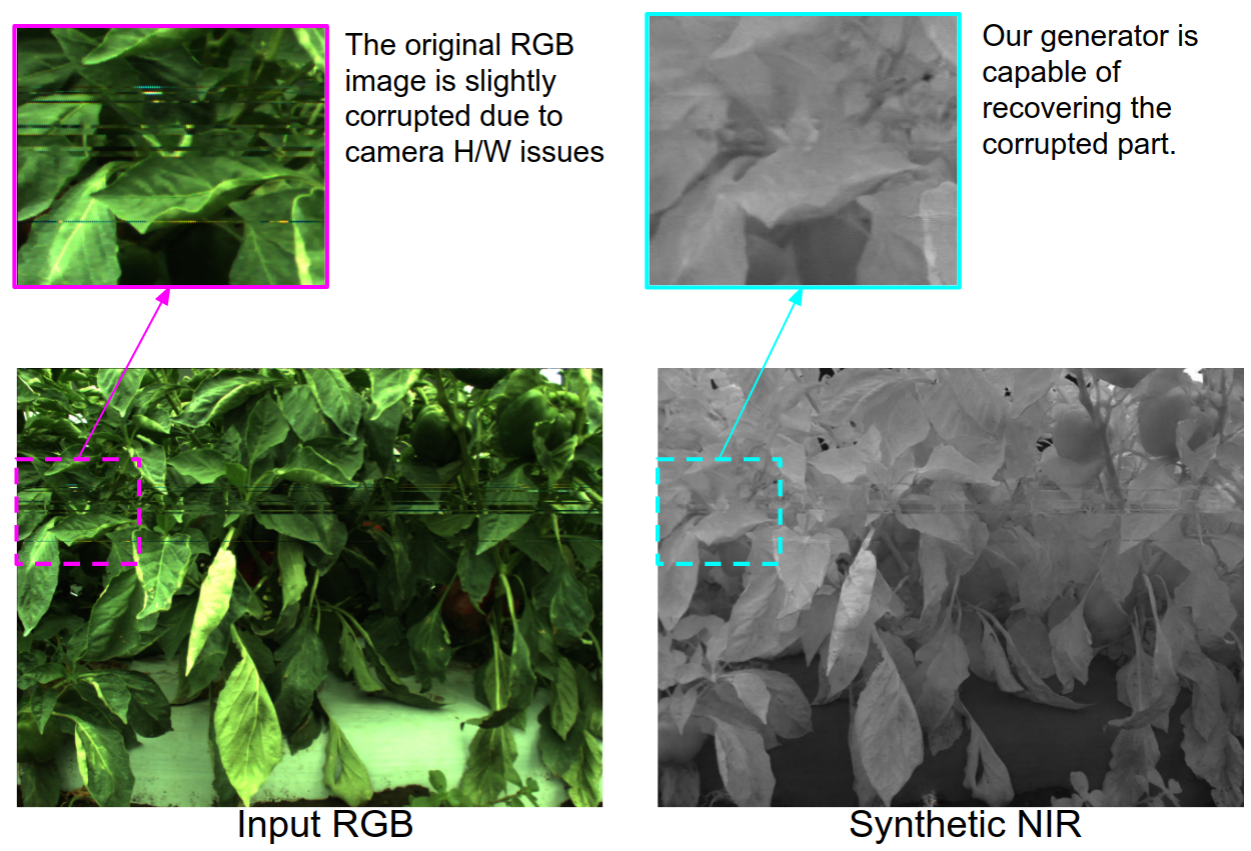
This figure illustrates in-painting capabilities of our generator. The left image shows data corruption due to hardware issue and the right depicts recovered region of interest (cyan) in synthetic NIR image.

While conducting the experiments, we observed interesting points and limitations of the proposed approach. Firstly, our synthetic NIR generator can recover small defected data. As shown in Figure 15, there were a couple of corrupted horizontal lines due to camera hardware issues (e.g., data stream reaches maximum bandwidth of the ethernet interface or abnormally high camera temperature). These artefacts are slightly recovered in the Synthetic NIR because the generator learnt how to incorporate adjacent pixel information to determine the NIR pixel value. Eventually, this creates a blurring effect, filling one horizontal line with interpolated data. We agree that it is difficult to argue whether this leads positive or negative impact on the performance. However, if the level of corruption is small (e.g., one or two-pixel rows) and frequently happens, the generator can effectively reject the abnormality.

**Figure 16:**
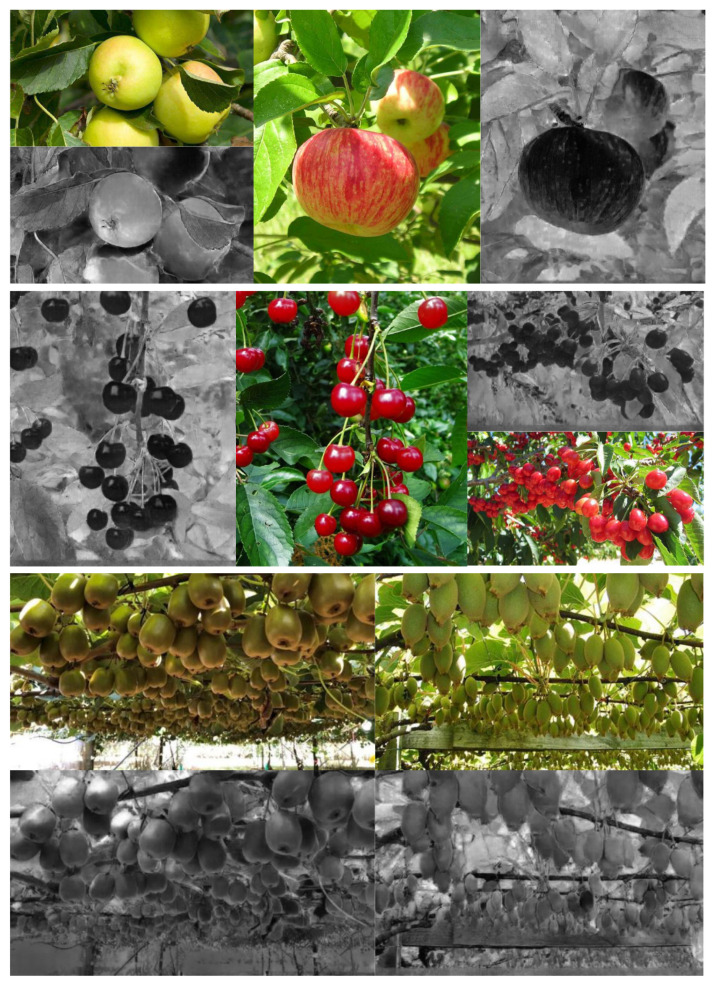
Synthetic NIR generation other than capsicum dataset. Each row shows the results for apple, cherry and kiwi, respectively. Apple detection performance improved 0.005 ${\mathrm{mAP}}_{\left[0.5:0.95\right]}$ with synthetic NIR. Whereas cherry and kiwi’s performance decreased by 0.03 and 0.09 ${\mathrm{mAP}}_{\left[0.5:0.95\right]}$.

Another discussion point and limitation is a marginal improvement or even degraded performance with synthetic NIR images. Only 4 fruits out of 11 demonstrated superior results with the additional information. The major reason is the large discrepancy between train and test sets. For example, Figure 16 shows synthetic NIR images given RGB inputs for apple, cherry and kiwi. Moreover, these test images were obtained from the Internet to hold high variation properties. Therefore, it is difficult to tell if the generated images are good or poor due to the lack of original NIR images.

**Figure 17:**
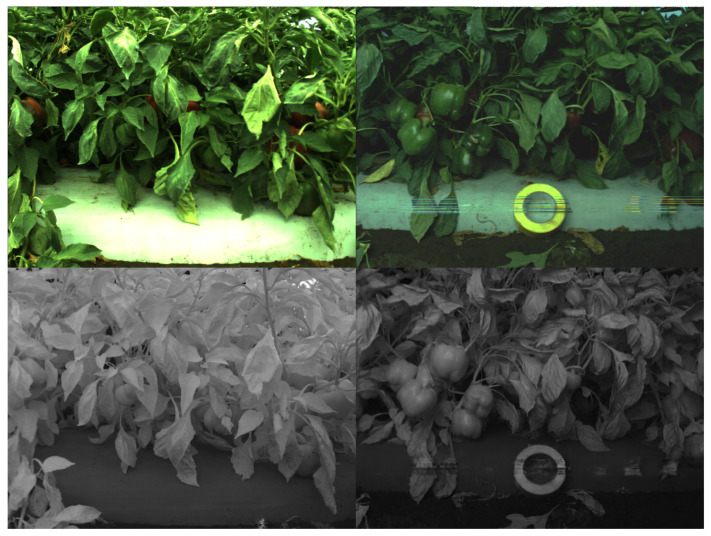
Synthetic NIR generation of capsicum dataset. Each row shows RGB and the corresponding NIR. Interestingly, capsicum detection performance decreased 0.01 ${\mathrm{mAP}}_{\left[0.5:0.95\right]}$ with synthetic NIR but increased 0.01 ${\mathrm{mAP}}_{\left[0.5\right]}$. This suggests that synthetic NIR contributed to inaccurately detect instances IoU<0.5 which can be hidden capsicums under shadow. However, these are rejected in evaluating the more strict metric, ${\mathrm{mAP}}_{\left[0.5:0.95\right]}$.

On the other hand, as shown in Figure 17, our generator properly produced synthetic NIR images given test images sampled from a similar distribution of train set. From this experiment, we would like to argue that it is very challenging to generalise our generator model, resulting in faulty and unrealistic samples. However, it should work with samples drawn from similar environments and conditions. Critical properties are consistent lighting and radiometric calibration.

## 6. Conclusions and Outlook

In this paper, we presented methodologies for generating synthetic NIR images using deep neural networks un-supervised (only required NIR–RGB pair). By adopting three public datasets with oversampling, we demonstrated the importance of the scale of the training dataset. It turned out that even with redundant information, it helped to stabilise parameters and led to superior performance. We re-processed these datasets and made them publicly available. These synthetic NIR images are rigorously evaluated with 11 fruits (seven from our previous study and four newly added dataset). These are also publicly available in various bounding box formats. This will allow other researchers to use this dataset easily and in a timely way. Early-fusion manner object detection experiments are conducted, and detailed analysis and discussion are shared with readers.

Although the scale of object annotation is relatively smaller than other giant datasets such as ImageNet, COCO or KITTI, these agriculture and horticulture-focused datasets will be useful in many aspects. It can be used for model in-domain-pretraining, a pre-step prior to in-task training (or finetune). For instance, if one wants to train a cherry detector with its own dataset, it makes more sense to pretrain with our dataset rather than ImageNet or COCO, which contain a lot of non-agricultural contexts (e.g., car, buildings, motorcycle, or ship). Another use case is that this small dataset can generate pseudo annotations. Given an unannotated dataset (e.g., 100 k kiwi images), one can obtain predictions (i.e., bounding boxes with confidences) using a trained model on a small dataset. Recursive iterations can improve performance by a large margin over a baseline model [53].

To our best knowledge, this paper introduces the most varied type of fruit/crop bounding box annotation dataset at the moment of writing and we hope this is useful for other follow-up studies.

## Figures and Tables

**Table 1:** Summary of related studies on NIR–RGB dataset.

Study	Advantages	Can Improve
Brown et al. [13]	Outdoor daily-life scenes	A temporal discrepancy in a pair Lack of radiometric calibration Small scale dataset to use
Chebrolu et al. [16]	Comprehensive large-scale dataset	Lack of comprehensive dataset summary table
Schmitt et al. [14]	Sampling from different locations over 4 seasons	Requires preprocessing to use

**Table 2:** Summary of datasets used for generating synthetic images.

Name	Desc.	# Train	# Valid	# Test	Total	Img Size (wxh)	Random Cropping	Over Sampling	Spectral Range (nm)
nirscene1		2880	320	320	3520	256 × 256	Yes	×10	400–850
		14,400	1600	1700	17,700	256 × 256	Yes	×100	
		28,800	3200	3400	35,400	256 × 256	Yes	×200	
		57,600	6400	6800	70,800	256 × 256	Yes	×400	
SEN12MS	All seasons	144,528	18,067	18,067	180,662	256 × 256	No	N/A	450–842
	Summer	36,601	4576	4576	45,753	256 × 256	No	N/A	
capsicum		1291	162	162	1615	1280 × 960	No	N/A	400–790

**Table 3:** Dataset for object detection summary table.

Name	Train (80%)	# Images Valid (10%)	Test (10%)	Total	# Instances	Median Image Ratio (wxh)
apple	49	7	7	63	354	852 × 666
avocado	67	7	10	84	508	500 × 500
blueberry	63	8	7	78	3176	650 × 600
capsicum	98	12	12	122	724	1290 × 960
cherry	123	15	16	154	4137	750 × 600
kiwi	100	12	13	125	3716	710 × 506
mango	136	17	17	170	1145	500 × 375
orange	52	6	8	66	359	500 × 459
rockmelon	77	9	11	97	395	1290 × 960
strawberry	63	7	9	79	882	800 × 655
wheat	2699	337	337	3373	147,793	1024 × 1024

**Table 4:** Train/inference summary table.

Type	Name	Desc.	GPU	Epoch	Train (h)	Test (ms/Image)	Img Size	# Samples
Synthetic image generation	nirscene	10×	P100	300	8	500	w:256 h:256	2880
		100×		300	72			14,400
		200×		300	144			28,800
		400×		164	113			57,600
	SEN12MS	All	RTX 3090	112	58	250	w:256 h:256	144,528
		Summer		300	140			36,601
	Capsicum		RTX 3090	300	29	780	w:1280 h:960	129
Fruit detection	10 Fruits	Yolov5s	RTX 3090	600	min: 4 min, max: 36 min	4.2–10.3	w:640 h:640	<5000
		Yolov5x						
	Wheat	Yolov5s	RTX 3090		1 h 16 min	4.2–10.3	w:640 h:640	147,793
		Yolov5x			2 h 16 min			

**Table 5:** Synthetic image generation quantitative results.

	FID ↓	# Train	# Valid	# Test	Img Size	Desc.
[7]	28	3691	N/A	N/A	256 × 256	10 layer attentions, 16 pixels, 4 layers, encoding+decoding
nirscene1	109.25	2880	320	320	256×256	×10 oversample
	42.10	14,400	1600	1700	256 × 256	×100 oversample
	32.10	28,800	3200	3400	256 × 256	×200 oversample
	27.660	57,600	6400	6800	256 × 256	×400 oversample, epoch 89
	**26.53**	86,400	9600	9600	256 × 256	×400 oversample, epoch 114
SEN12MS	16.47	36,601	4576	4576	256 × 256	Summer, 153 epoch
	**11.36**	144,528	18,067	18,067	256 × 256	All season, 193 epoch
capsicum	40.15	1102	162	162	1280 × 960	150 epoch

**Table 6:** Eleven fruits/crops object detection quantitative results table. Up-arrow indicates a higher score is better performance. Bold denotes the best performance in the corresponding metric within each fruit.

Name	Model	Input	${\mathrm{mAP}}_{0.5}$↑	${\mathrm{mAP}}_{\left[0.5:0.95\right]}$↑	Precision↑	Recall↑	F1↑
apple	yolov5s	RGB	0.9584	0.7725	0.9989	0.913	0.9540
		RGB+NIR	**0.9742**	**0.7772**	0.9167	0.9565	0.9362
	yolov5x	RGB	0.9688	0.7734	0.9989	0.9130	0.9540
		RGB+NIR	0.9702	0.7167	0.8518	0.9999	0.9199
avocado	yolov5s	RGB	0.8419	0.4873	0.9545	0.8077	0.8750
		RGB+NIR	0.8627	0.4975	0.8749	0.8071	0.8396
	yolov5x	RGB	**0.9109**	0.6925	1.0000	0.8461	0.9166
		RGB+NIR	0.8981	**0.6957**	0.9583	0.8846	0.9200
blueberry	yolov5s	RGB	**0.9179**	**0.5352**	0.8941	0.9018	0.8979
		RGB+NIR	0.8998	0.5319	0.9224	0.8354	0.8767
	yolov5x	RGB	0.9093	0.5039	0.9345	0.8494	0.8899
		RGB+NIR	0.8971	0.4657	0.9063	0.8476	0.8760
capsicum	yolov5s	RGB	0.8503	0.4735	0.8159	0.8473	0.8313
		RGB+NIR	0.8218	0.4485	0.848	0.8091	0.8281
	yolov5x	RGB	0.8532	**0.4909**	0.8666	0.8429	0.8546
		RGB+NIR	**0.8642**	0.4812	0.8926	0.8244	0.8571
cherry	yolov5s	RGB	0.9305	0.6045	0.9300	0.8586	0.8929
		RGB+NIR	0.9034	0.5655	0.8994	0.8505	0.8743
	yolov5x	RGB	**0.9415**	**0.6633**	0.929	0.8747	0.9010
		RGB+NIR	0.9300	0.6325	0.9129	0.8687	0.8903
kiwi	yolov5s	RGB	0.8642	0.5651	0.9196	0.7831	0.8459
		RGB+NIR	0.8154	0.5195	0.9039	0.7188	0.8008
	yolov5x	RGB	**0.8935**	**0.6010**	0.9056	0.8326	0.8676
		RGB+NIR	0.8240	0.5139	0.8625	0.7643	0.8104
mango	yolov5s	RGB	0.9431	0.6679	0.9516	0.879	0.9139
		RGB+NIR	0.9032	0.6033	0.9347	0.8217	0.8746
	yolov5x	RGB	**0.9690**	**0.6993**	0.9333	0.8917	0.9120
		RGB+NIR	0.9339	0.6681	0.9221	0.9044	0.9132
orange	yolov5s	RGB	0.9647	0.707	0.9409	0.9697	0.9551
		RGB+NIR	0.9488	0.7669	0.9655	0.8482	0.9031
	yolov5x	RGB	**0.9662**	**0.8484**	0.9998	0.9091	0.9523
		RGB+NIR	0.9584	0.8277	1.0000	0.9091	0.9524
rockmelon	yolov5s	RGB	0.9588	0.6321	0.9198	0.8846	0.9019
		RGB+NIR	0.9205	0.6701	0.9999	0.8462	0.9167
	yolov5x	RGB	**0.9612**	**0.7161**	0.9259	0.9615	0.9434
		RGB+NIR	0.9444	0.7018	0.8926	0.9615	0.9258
strawberry	yolov5s	RGB	**0.9553**	**0.6995**	0.9559	0.8784	0.9155
		RGB+NIR	0.8913	0.6210	0.9000	0.8513	0.8750
	yolov5x	RGB	0.8899	0.5237	0.8954	0.8108	0.8510
		RGB+NIR	0.9071	0.4882	0.9375	0.8106	0.8694
wheat	yolov5s	RGB	0.9467	0.5585	0.929	0.9054	0.9170
		RGB+NIR	0.9412	0.5485	0.9258	0.8926	0.9089
	yolov5x	RGB	**0.9472**	**0.5606**	0.9275	0.9035	0.9153
		RGB+NIR	0.9294	0.5329	0.9163	0.9005	0.9083

## Data Availability

Not applicable.

## Supplementary Materials

The following are available at https://www.mdpi.com/article/10.3390/s22134721/s1, Figure S1: Apple prediction results using Yolov5x and RGB test images that achieved 0.78 ${\mathrm{mAP}}_{\left[0.5:0.95\right]}$. Images are obtained from [15]; Figure S2: Avocado prediction results using Yolov5x and RGB test images that achieved 0.77 ${\mathrm{mAP}}_{\left[0.5:0.95\right]}$. Images are obtained from [15]; Figure S3: Blueberry prediction results using Yolov5x and RGB test images that achieved 0.50 ${\mathrm{mAP}}_{\left[0.5:0.95\right]}$. Blueberry images have relatively high instances/image ratio which yields lower mAP. Images are obtained from Google Images; Figure S4: Capsicum prediction results using Yolov5x and RGB test images that achieved 0.49 ${\mathrm{mAP}}_{\left[0.5:0.95\right]}$. This is one of the most challenging dataset that collected complex and cluttered real farm environments. Lighting condition is severe, level of occlusion is high, and distance to objects is far. Images are obtained from [15]; Figure S5: Cherry prediction results using Yolov5x and RGB test images that achieved 0.66 ${\mathrm{mAP}}_{\left[0.5:0.95\right]}$. Images are obtained from Google Images; Figure S6: Kiwi prediction results using Yolov5x and RGB test images that achieved 0.60 ${\mathrm{mAP}}_{\left[0.5:0.95\right]}$. Images are obtained from Google Images; Figure S7: Mango prediction results using Yolov5x and RGB test images that achieved 0.69 ${\mathrm{mAP}}_{\left[0.5:0.95\right]}$. Images are obtained from [15]; Figure S8: Orange prediction results using Yolov5x and RGB test images that achieved 0.73 ${\mathrm{mAP}}_{\left[0.5:0.95\right]}$. Images are obtained from [15]; Figure S9: Mango prediction results using Yolov5x and RGB test images that achieved 0.69 ${\mathrm{mAP}}_{\left[0.5:0.95\right]}$. Top row images are obtained from [15] and bottom are from Google Images; Figure S10: Strawberry prediction results using Yolov5x and RGB test images that achieved 0.67 ${\mathrm{mAP}}_{\left[0.5:0.95\right]}$. Images are obtained from [15]; Figure S11: Wheat prediction results using Yolov5x and RGB test images that achieved 0.56 ${\mathrm{mAP}}_{\left[0.5:0.95\right]}$ Images are obtained from Kaggle wheat detection competition.

## Abbreviations

The following abbreviations are used in this manuscript:

NIR	Near-Infrared
GPU	Graphics Processing Unit
DN	Digital Number
FID	Frechet Inception Distance
mAP	Mean Average Precision
AP	Average Precision
ML	Machine Learning
NLP	Natural Language Processing
NDVI	Normalised Difference Vegetation Index
NDWI	Normalised Difference Water Index
EVI	Enhanced Vegetation Index
LiDAR	Light Detection and Ranging
RGB-D	Red, Green, Blue and Depth
RTK-GPS	Real-Time Kinematic Global Positioning System
ESA	European Space Agency
GeoTIFF	Geostationary Earth Orbit Tagged Image File Format
GAN	Generative Adversarial Networks
cGAN	Conditional Generative Adversarial Networks
Pix2pix	Pixel to pixel
OASIS	You Only Need Adversarial Supervision for Semantic Image Synthesis
MRI	Magnetic Resonance Image
CTI	Computed Tomography Image
UNIT	Unsupervised Image-to-Image Translation Network
RPN	Region Proposal Network
DNN	Deep-Neural Networks
GDS	Ground Sample Distance
YOLOv5	You Only Look Once
BN	Batch-Normalisation
ReLU	Rectified Linear Unit
CBL	Convolution Batch Normalisation and Leaky ReLU
CSP	Cross Stage Partial
SPP	Spatial Pyramid Pooling
FPN	Feature Pyramid Networks
PAN	Path Aggregation Network
SSD	Single Stage Detectors
GIoU	Generalised Intersection Over Union
IoU	Intersection Over Union
COCO	Common Objects in Context
GAM	Generative Adversarial Metric
TP	True Positive
TN	True Negative
FP	False Positive
FN	False Negative
P	Precision
R	Recall
MAE	Mean Absolute Error
SSIM	Structural Similarity
