# Supplementary material for: deepNIR: Datasets for Generating Synthetic NIR Images and Improved Fruit Detection System Using Deep Learning Techniques
Source: Sensors (Basel). 2022 Jun 22;22(13):4721. doi: 10.3390/s22134721 (PMC9269522; doi:10.3390/s22134721)
Supplement: Supplementary file 1 [file sensors-22-04721-s001.zip › sensors-1713406-supplementary.pdf]

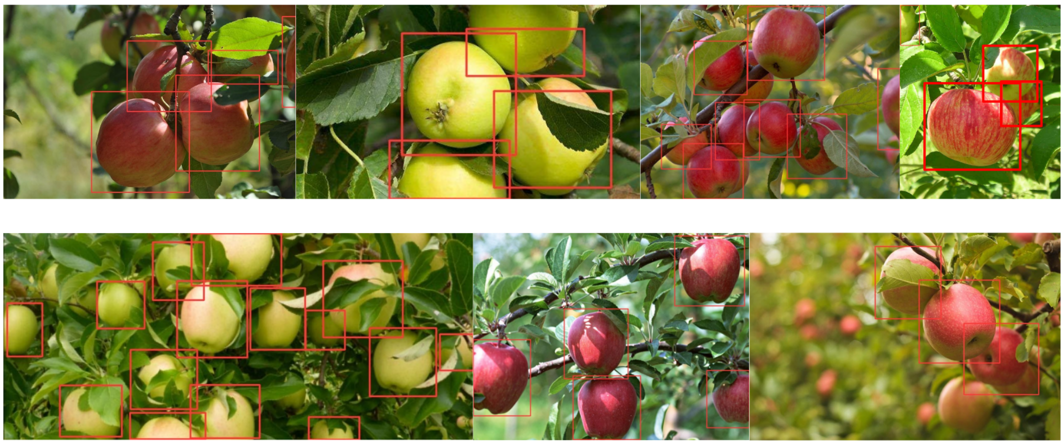

Figure S1. Apple prediction results using Yolov5x and RGB test images that achieved 0.78 $mAP_{[0.5:0.95]}$. Images are obtained from [15].

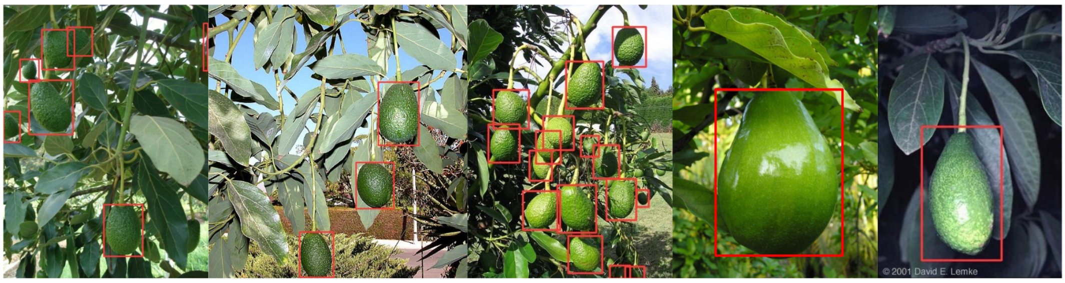

Figure S2. Avocado prediction results using Yolov5x and RGB test images that achieved 0.77 $mAP_{[0.5:0.95]}$. Images are obtained from [15].

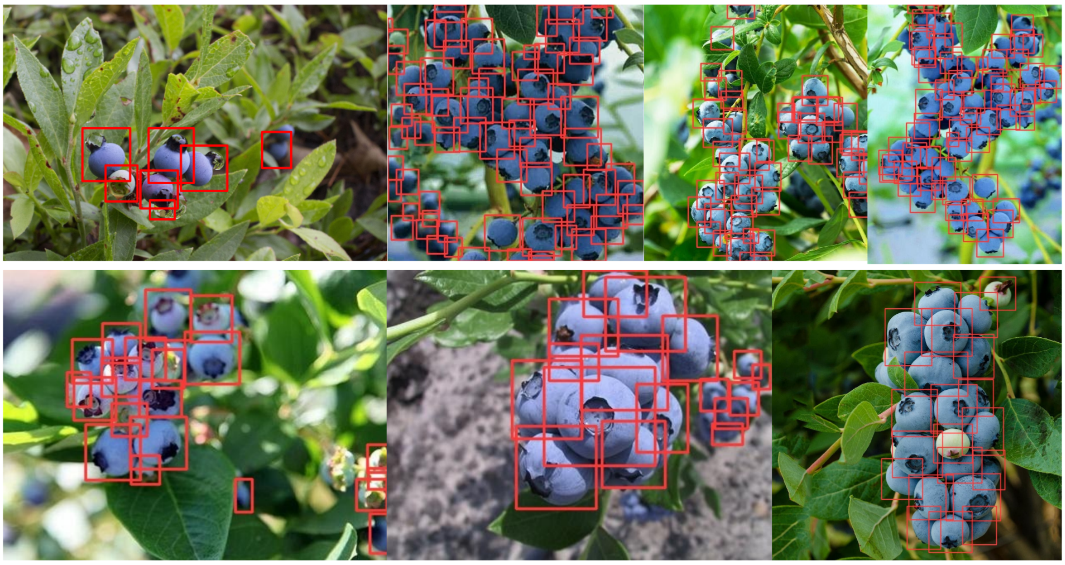

Figure S3. Blueberry prediction results using Yolov5x and RGB test images that achieved 0.50 $mAP_{[0.5:0.95]}$. Blueberry images have relatively high instances/image ratio which yields lower mAP. Images are obtained from Google Images.

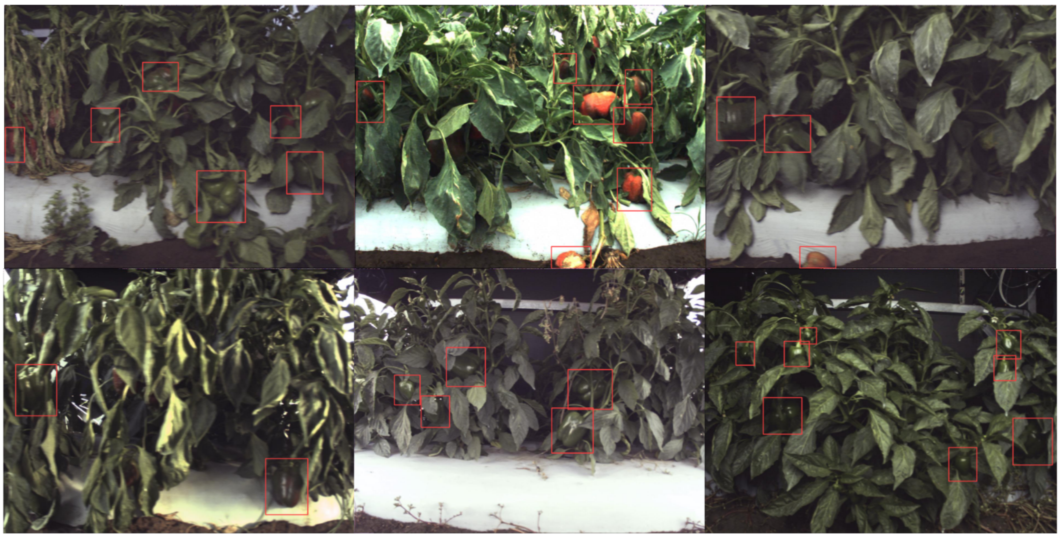

Figure S4. Capsicum prediction results using Yolov5x and RGB test images that achieved 0.49 $mAP_{[0.5:0.95]}$. This is one of the most challenging dataset that collected complex and cluttered real farm environments. Lighting condition is severe, level of occlusion is high, and distance to objects is far. Images are obtained from [15].

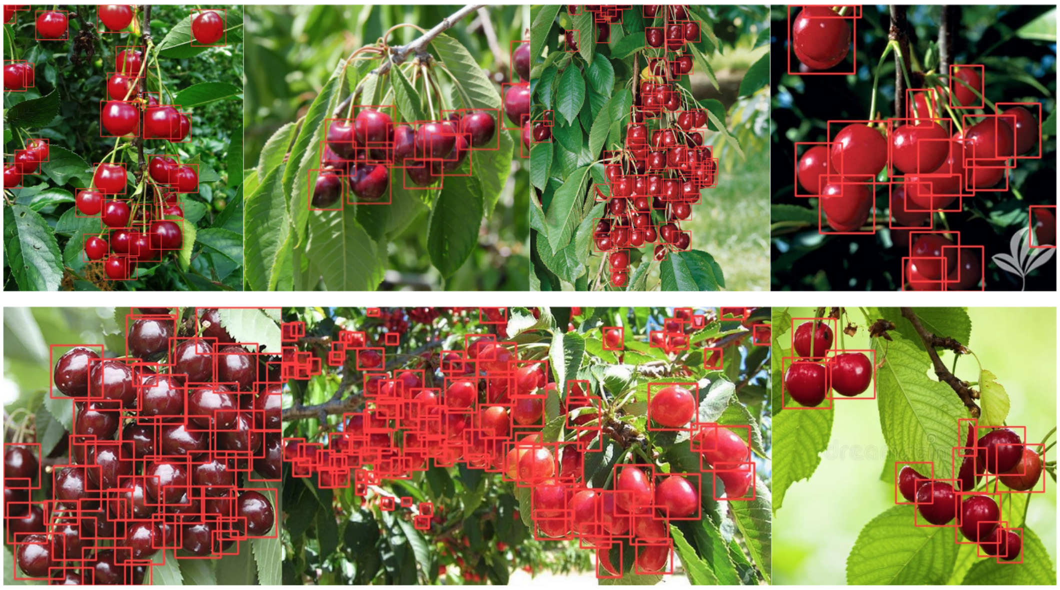

Figure S5. Cherry prediction results using Yolov5x and RGB test images that achieved 0.66 $mAP_{[0.5:0.95]}$. Images are obtained from Google Images.

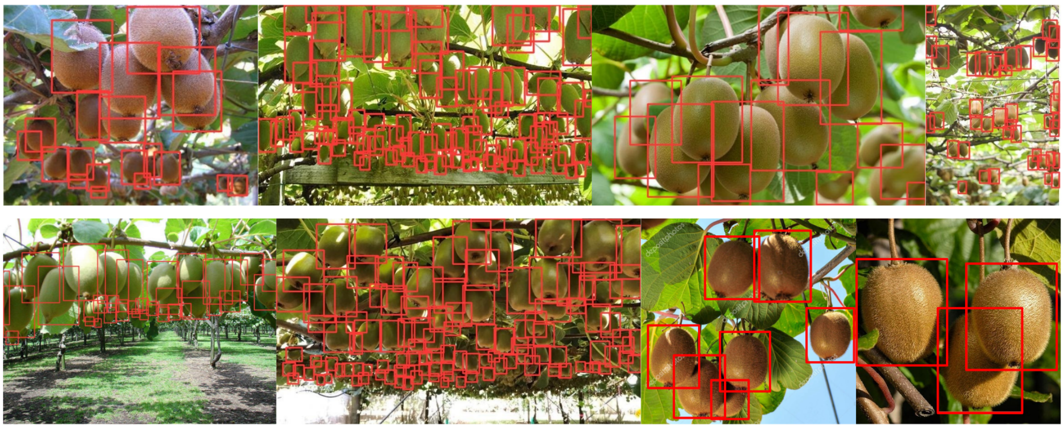

Figure S6. Kiwi prediction results using Yolov5x and RGB test images that achieved 0.60 $mAP_{[0.5:0.95]}$. Images are obtained from Google Images.

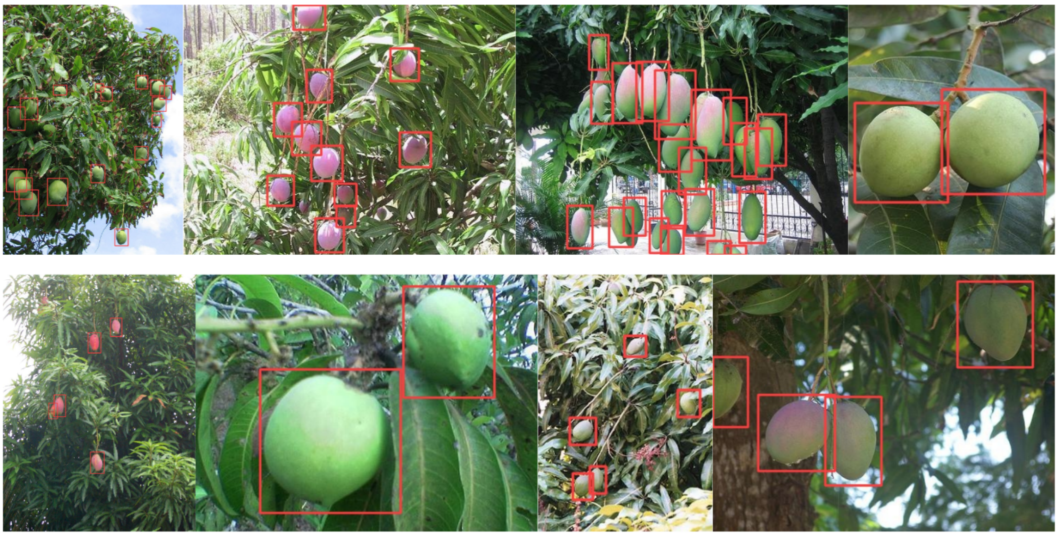

Figure S7. Mango prediction results using Yolov5x and RGB test images that achieved 0.69 $mAP_{[0.5:0.95]}$. Images are obtained from [15].

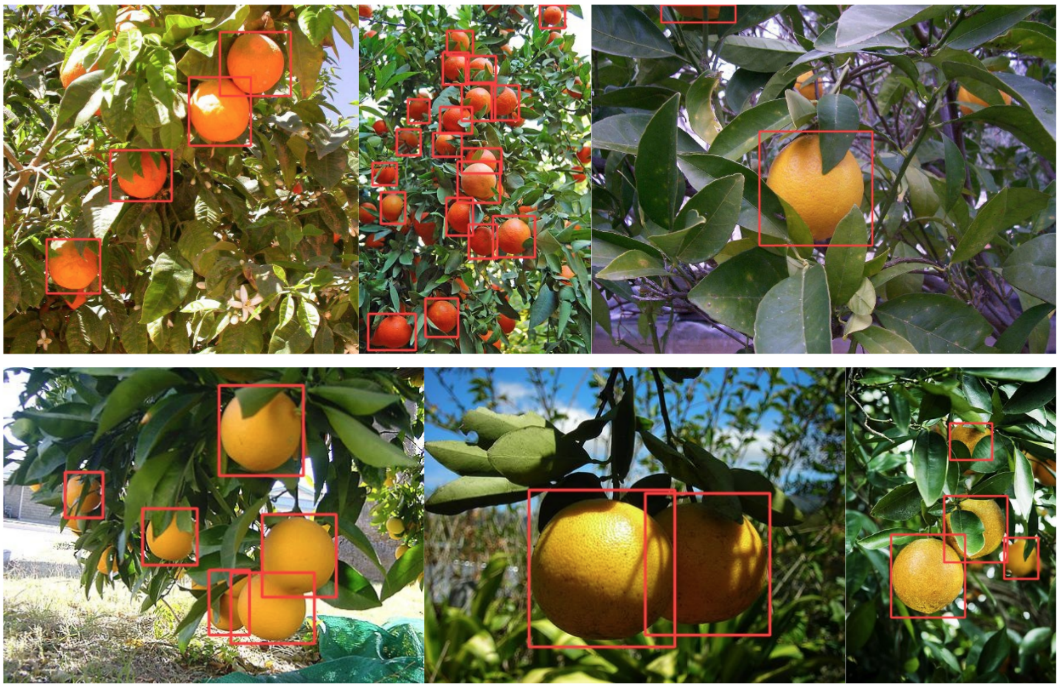

Figure S8. Orange prediction results using Yolov5x and RGB test images that achieved 0.73 $mAP_{[0.5:0.95]}$. Images are obtained from [15].

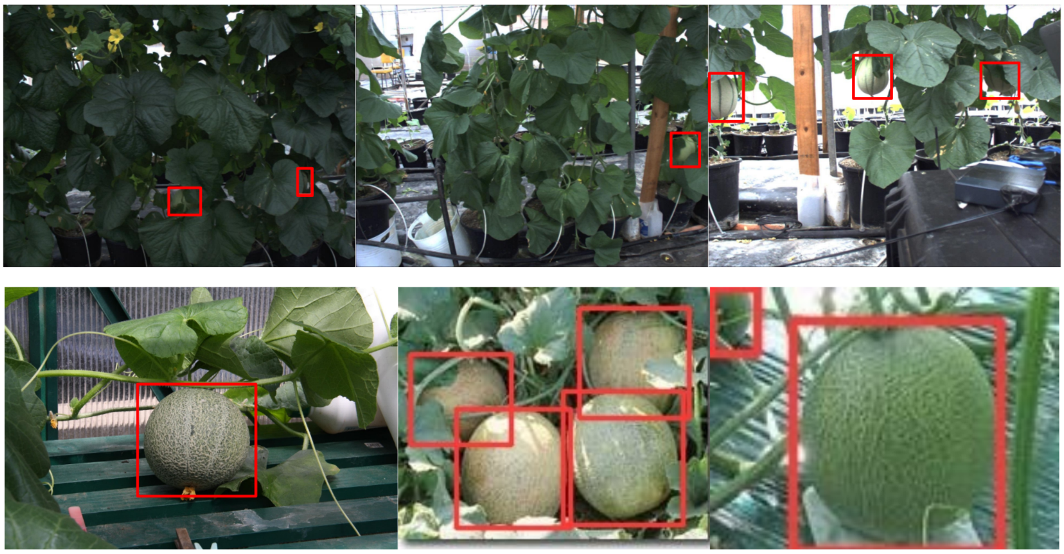

Figure S9. Mango prediction results using Yolov5x and RGB test images that achieved 0.69 $mAP_{[0.5:0.95]}$. Top row images are obtained from [15] and bottom are from Google Images.

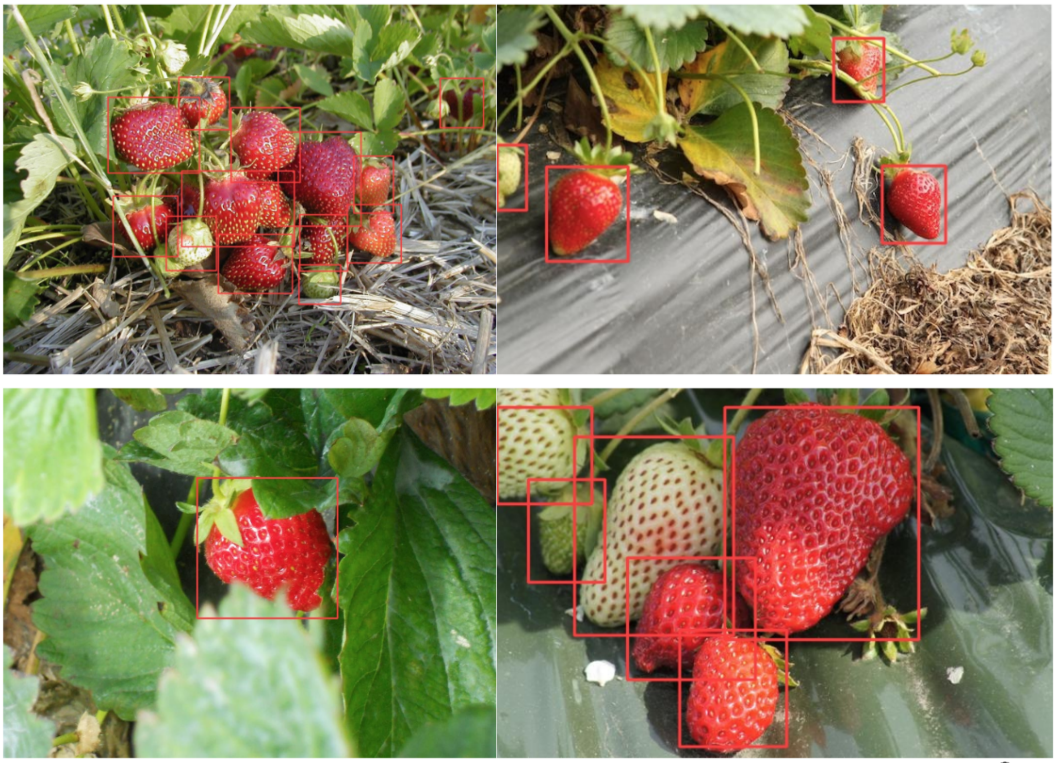

Figure S10. Strawberry prediction results using Yolov5x and RGB test images that achieved 0.67 $mAP_{[0.5:0.95]}$. Images are obtained from [15].

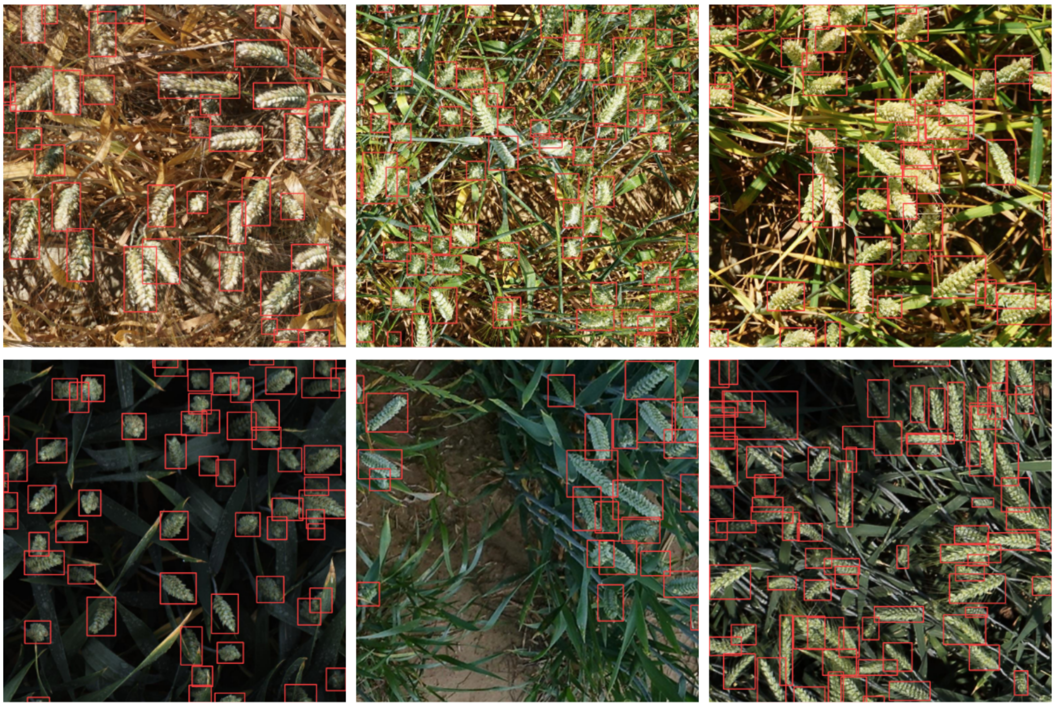

Figure S11. Wheat prediction results using Yolov5x and RGB test images that achieved 0.56 $mAP_{[0.5:0.95]}$ Images are obtained from Kaggle wheat detection competition.
